# Event-triggered iterative learning control for output constrained multi-agent systems

**DOI:** 10.1371/journal.pone.0315209

**Published:** 2025-03-21

**Authors:** Wei Cao, Huanhuan Li, Jinjie Qiao, Yi Zhu

**Affiliations:** 1 College of Computer and Control Engineering, Qiqihar University, Qiqihar, China; 2 College of Economics and Management, Qiqihar University, Qiqihar, China; Cyprus International University Faculty of Engineering: Uluslararasi Kibris Universitesi Muhendislik Fakultesi, TÜRKIYE

## Abstract

An event-triggered iterative learning consensus tracking control strategy is proposed for output constrained nonlinear discrete-time multi-agent systems. Firstly, the estimated Pseudo partial derivative(PPD) algorithm is determined based on the input and output data of the system, and the output observer is designed based on the estimated PPD. Secondly, the deadband controller is designed based on the output estimation error of the observer, and the event trigger condition is determined by comparing the size of the output estimation error and the deadband controller function value, and the agents communicate when the trigger condition is satisfied, and do not communicate when it is not satisfied. Then, the event-triggered iterative learning control algorithm is constructed using the estimated PPD, the trigger condition and the measurement error, and the convergence of the algorithm is proved by using the Lyapunov function, and the proposed algorithm can make the output constrained multi-agent system consistently and completely tracking on the desired trajectory without the need of real-time communication conditions. Finally, the simulation results further validate the effectiveness of the control protocol.

## 1. Introduction

A multi-agent system is a system that consists of a number of individual agents with sensing and execution capabilities, and that accomplishes a complex task through inter-agent coordination. Multi-agent systems [1] are autonomous, fault-tolerant, collaboratively distributed and scalable, and have higher performance and efficiency than single systems. Currently, many experts in the field of control have conducted extensive research on multi-agent systems. For example, the cluster control problem [2,3] the consensus problem [4–7], and the formation control problem [8–10] for agent bodies. Among them, consensus is the basis for the study of other problems in multi-agent systems. Consensus of a multi-agent system refers to the convergence of one or some states of all the agents in the network [11–13]. Most of the research results only achieve consensus of the agents in the time domain, i.e., the states and outputs of the system converge as time approaches infinity. However, in practice, there exists a class of control systems that perform specific tasks repeatedly or periodically for a finite period time. For such systems, conventional control algorithms are no longer applicable.

Iterative learning control is an effective control method to achieve complete tracking over a finite time interval [17–22]. The algorithm is suitable for systems with repetitive operation characteristics, and the controller structure of the algorithm is simple and has low requirements on the system model. In view of these advantages, many scholars have studied the consensus problem of multi-agent systems using iterative learning control methods. For example, [23] used an iterative learning control scheme to study the consensus problem of discrete linear multi-agent systems. In [24], an iterative learning algorithm is proposed to be unified in both continuous and discrete time domains, which can ensure that the output of the system converges to the desired trajectory within a finite time interval. Therefore, [25] investigated the consensus problem of a class of nonlinear multi-agent systems, and utilized an iterative learning control algorithm to achieve full tracking of the desired trajectory. In [26], D-type and PD-type learning laws with iterative initial states are used to solve the consensus tracking problem of nonlinear multi-agent systems with impulsive inputs. In [27], an adaptive iterative learning control method is investigated to solve the consensus problem of nonlinear multi-agent systems under state constrained. In [28], an iterative learning control method is adopted to solve the consensus problem of continuous linear multi-agent systems under output constrained, and the convergence of the system is proved by using the norm and disk theorem. In [29], a distributed iterative learning control algorithm is proposed to solve the consensus problem of discrete nonlinear multi-agent systems with output constrained.

Based on the above literature, considering saving system resources and reducing computer energy loss, many scholars in recent years have proposed an event-triggered iterative learning control strategy for the consensus problem of multi-agent system. For example, [30] proposed an event-triggered iterative learning control method for the consistency problem of nonlinear discrete-time multi-agent systems. In [31], an event-triggered distributed model-free iterative learning control strategy is proposed for the consensus problem of nonlinear multi-agent systems with random link packet loss. The above control algorithm can indeed reduce system resources and computer energy loss, but it is no longer suitable for solving the saturation problem of actuators and sensors in the communication process. For this reason, [32] proposed an event-triggered control protocol to study the consensus problem of nonlinear multi-agent systems with relative state constrained. In [33], static and dynamic event-triggered strategies are proposed to deal with the multi-agent consensus problem with output constrained.

Analyzing the above literature, it is found that [23,24,28] studied the consensus problem of linear multi-agent system by using the iterative learning control method, compared with the linear system, in the practical application, the state variables of the system are mostly nonlinear relationships between them, so the above control algorithms are not applicable for the nonlinear strongly coupled system. In [30,31], although the event-triggered iterative learning control algorithm is used to solve the problem of complete tracking in a finite time, the control algorithm did not take into account the fact that the output is limited, which makes the system unstable or even destabilized. The control algorithms in [32,33] solved the problem of consistent tracking and space resource saving for the system under output constrained, but the algorithms realized the goal of trajectory tracking when the time tends to infinity, and did not consider the problem of repeated operation of trajectory tracking for a finite period of time. [29] used iterative learning control algorithm to solve the consistency problem of the system under output constrained, but [29] did not consider the problem of insufficient system space and bandwidth constraints, and when the system is in the ideal state and still receive and send data periodically, which will be a large amount of waste of space resources and computer energy loss.

In view of the above analysis, in order to make the output constrained nonlinear multi-agent systems realize consistent and complete tracking in a limited time, and analyzing it from the perspective of saving the system resources and computer energy loss, an event-triggered distributed iterative learning control algorithm is proposed. The main contributions of this method are reflected in the following three points: 1) The time-varying gain of this control algorithm consists of PPDs, and the tracking error percentage can be adjusted in real time to control the input data changes. The control algorithm does not need a specific mathematical model of the controlled object, and has few parameters and a simple structure. 2) Give the sufficient condition for system convergence, design the output observer by using PPD, and monitor the output data changes in real time to solve the consensus convergence problem of the system under the output constrained. 3) Design the dead zone controller to avoid Zeno phenomenon, by comparing the output estimation error and the size of the dead zone controller function value, to design the event triggered mechanism, under the triggered mechanism, each agent only needs to judge whether to communicate or not according to its own triggered conditions, to avoid the interference of the communication between the agents to affect the triggered conditions. At the same time, the algorithm reduces the energy loss and saves the system resources.

This paper is organized as follows: section 2 introduces the main contents of the study, including graph theory knowledge, dynamic model description of agents and output constrained description; section 3 is about the controller design, including the definition of event-triggered PPD, the design of the event communication mechanism, the design of the controller, as well as the proof of convergence of the algorithms and the theorems; section 4 verifies the effectiveness of the proposed algorithms through numerical simulation; section 5 summarizes and gives an outlook for future research.

## 2. Problem description

### 2.1. Preliminaries

The communication topology between agents in a multi-agent system can be described by a graph theory, therefore, the graph *G* is defined as $G=\left(V,E,A\right)$ to represent the communication topology between agents, where: *V*, *E*, and *A* respectively are the vertex set, the edge set and the adjacency matrix of the graph, V={1,2,…,N}, E⊆$\left\{\left(i,j\right)|i,j\in V\right\}$⊆V×V, $A=\left[a_{ij}\right]\in R^{N\times N}$. The element $\left(i,j\right)$ in *E* indicates that the agents i, j can communicate with each other, $a_{ij}\in \left\{0,1\right\}$ is the weight value of the edge, if agent *j* can receive information from agent *i*, then $a_{ij}=1$; if agent *j* cannot receive information from agent *i*, then $a_{ij}=0$. The set of neighboring of an agent is $N_{j}=\left\{j\in V|\left(j,i\right)\in E\right\}$, and the degree matrix is $\bar{D}=diag \{{\bar{d}}_{1},{\bar{d}}_{2},$$\left.\ldots ,{\bar{d}}_{N}\right\}$, ${\bar{d}}_{j}=\sum_{i=1}^{N}a_{ij}$ is the sum of the elements of the *j*th row of the adjacency matrix *A*, and the Laplace matrix of the graph *G* is represented as $L=\bar{D}-A$. The complete topology with virtual leader 0 can be described as $\bar{G}=\left(V\cup \left\{0\right\},\bar{E},\bar{A}\right)$, $\bar{E}$ denotes the set of edges of the graph $\bar{G}$, and $\bar{A}$ denotes the adjacency matrix of the graph $\bar{G}$. Consider the relationship between a virtual leader and a follower, if the agent *j* is directly connected to the virtual leader, then $d_{j}=1$; otherwise $d_{j}=0$, and the matrix is denoted as D=$diag\left\{d_{1},d_{2},\ldots ,d_{N}\right\}$.

### 2.2. Model statement

In this paper, we study a class of nonlinear discrete-time multi-agent systems consisting of *N* agents, where the dynamics of the *j*th agent is described as follows:


$$\{\begin{array}{l} x_{k,j}\left(t+1\right)=f\left(k,x_{k,j}\left(t\right)\right)+Bu_{k,j}\left(t\right)\\ y_{k,j}\left(t\right)=Cx_{k,j}\left(t\right)\\ z_{k,j}\left(t\right)=sat_{y_{0j}}\left(y_{k,j}\left(t\right)\right) \end{array}$$ (1)

where $t\in \left[0,1,\ldots ,T\right]$ is the time interval, j∈1,2,…,N denotes the *j*th agent, and *k* is used to represent the *k* th iteration, $x_{k,j}\in R^{n}$, $u_{k,j}\in R^{p}$, $y_{k,j}\in R^{m}$ respectively are the state vectors, control input vectors and measurement output vectors of the system, *B* is the input matrix of the system, and $f\left(.,.\right)$ is an unknown nonlinear function. $y_{0j}$ is the output constrained threshold of the *j*th agent $\left(y_{0j}>0\right)$, the output constrained value of each agent is expressed as $y_{0}=\left[y_{01}y_{02}\cdots y_{0N}\right]\in R^{N}$, the output constrained function is defined as follows:


$$z_{k,j}\left(t\right)=sat_{y_{0j}}\left(y_{k,j}\left(t\right)\right)$$

$$= \{\begin{array}{c} -y_{0j},y_{k,j}\left(t\right)\leq -y_{0j}\\ y_{k,j}\left(t\right),-y_{0j}<y_{k,j}\left(t\right)<y_{0j}\\ y_{0j},y_{k,j}\left(t\right)\geq y_{0j} \end{array}$$ (2)

For the purpose of this analysis, it is assumed that the system satisfies the following conditions:

**Assumption 1** [11] $f\left(.,.\right)$ is a continuous nonlinear function, and the partial derivative with respect to $u_{j}\left(k,t\right)$ exists.

**Assumption 2** [14–16] Let the system satisfy the generalized Lipschitz continuity condition along the iteration axis, i.e., there exists a r>0 such that $\left|\Delta z_{j}\left(k,t+1\right)\right|\leq r\left|\Delta u_{j}\left(k,t\right)\right|$, where $\Delta z_{j}\left(k,t+1\right)=z_{j}\left(k,t+1\right)-z_{j}\left(k-1,t+1\right)$; $\Delta u_{j}\left(k,t\right)$$=u_{j}\left(k,t\right)-u_{j}\left(k-1,t\right)$; and $\left|\left(\Delta u_{j}\left(k,t\right)\right)\right|\leq b$, b>0.

**Lemma 1** [14] The system can be represented by a dynamic linearized model in compact form, subject to assumptions 1 and 2, as follows:


$$\Delta z_{j}\left(k,t+1\right)=\Gamma_{j}(k,t)\Delta u_{j}(k,t)$$ (3)

where $\left|\Gamma_{j}(k,t)\right|\leq r$, $\Gamma_{j}(k,t)$ is the pseudo partial derivative, and time-varying, r>0.

**Assumption 3** [29] From the dynamic linearization model, $\Gamma_{j}(k,t)$ can be positive or negative, and in this paper we assume that $\Gamma_{j}(k,t)<0$. Define the consensus measurement error of the agent *j* at the *k*th iteration as:


$$\zeta_{j}\left(k,t\right)=\sum_{i\in N_{j}}a_{i,j}\left({\hat{z}}_{i}\left(k,t\right)-{\hat{z}}_{j}\left(k,t\right)\right)+$$

$$d_{j}\left(y_{d}\left(t\right)-{\hat{z}}_{j}\left(k,t\right)\right)$$ (4)

where ${\hat{z}}_{j}\left(k,t\right)$ is an estimate of $z_{j}\left(k,t\right)$. Let $e_{j}\left(k,t\right)=y_{d}\left(k\right)-y_{j}(k,t)$ denote the tracking error of the agent, the actual tracking error is $e_{j}^{\Xi }\left(k,t\right)=y_{d}\left(t\right)-z_{j}\left(k,t\right)=sat_{y_{d}\pm y_{0j}}\left(e_{j}\left(k,t\right)\right)$, define ${\hat{e}}_{j}\left(k,t\right)=y_{d}\left(t\right)-{\hat{z}}_{j}\left(k,t\right)$ to denote the estimation error of an agent, where $y_{d}\pm y_{0j}$ is the upper and lower bounds of the output constrained function.

**Definition 1** The multi-agent system is said to reach consensus if and only if the difference between the output trajectories of the agents and the desired trajectories is a small positive constant, i.e.


$$\underset{k\to \infty }{\lim}\left(y_{d}\left(t\right)-y_{j}\left(k,t\right)\right)=\iota$$ (5)

where j=1,2,…,N, 0<ι<0.02.

**Assumption 4** [29] The desired output $y_{d}\left(t\right)$ of the system at each moment in a finite time interval is within a measurable range, therefor $max\left(\left|y_{d}\left(t\right)\right|\right)<min(y_{01},y_{02},\ldots ,y_{0N})$. Define $e_{j}\left(t\right)= [e_{j,1}^{T},e_{j,2}^{T},\cdots ,$

${\left.e_{j,N}^{T}\right]}^{T}$, $e_{j}^{\Xi }\left(t\right)$$={\left[{e_{j1}^{\Xi }}^{T}\left(t\right),{e_{j2}^{\Xi }}^{T}\left(t\right),\ldots ,{e_{jN}^{\Xi }}^{T}\left(t\right)\right]}^{T}$, satisfying $0\leq {e_{j}^{\Xi }}^{T}(t)e_{j}\left(t\right)\leq e_{j}^{T}(t)e_{j}\left(t\right).$

**Assumption 5** In the graph $\bar{G}=\left(V\cup \left\{0\right\},\bar{E},\bar{A}\right)$, all the followers have direct or indirect access to the virtual leader’s trajectory information.

**Assumption 6** The initial state of the agent remains the same at each iteration, i.e., $x_{i,j}(0)=x_{j}^{0}$, $x_{j}^{0}$ is a given initial state.

**Remark 1** $\Gamma_{j}\left(k,t\right)<0$ is a condition for proving that ${\hat{\Gamma }}_{j}\left(k,t\right)$ is bounded.

**Remark 2** Assumption 5 is a necessary communication condition for achieving unified and complete tracking of multi-agent systems, if the agent in the system cannot directly or indirectly obtain the trajectory information of the virtual leader, then this agent will become an isolated agent in the system, unable to track the expected trajectory, and thus unable to achieve consensus tracking of the system.

**Remark 3** Assumption 6 is the basic condition for iterative learning control algorithm to achieve complete tracking of system trajectory, it indicates that the system strictly repeats the initial state each time it is tracked.

The control objective of this paper is to construct an event-triggered distributed iterative learning control algorithm for a nonlinear discrete-time multi-agent system satisfying assumptions 1–6 when the system output is constrained and when only some of the agents are able to acquire the desired trajectory information, so that all the agents can completely track the desired trajectory in a finite time interval.

For a better understanding of the meaning of the parameters mentioned below, the parameters are given in Table 1.

**Table 1:** Value of each parameter.

Parameter	Input the previous *b*	PPD previous *r*	Error previous *ι*	Step leader *ρ*	Weight *ϑ*
Value	b>0	r>0	0<ι<0.02	0<ρ<1	ϑ>0
Parameter	Observer feedback gain *χ*	*τ*	*u*	*λ*	Stabilizing weight *β*
Value	−1.5<χ<−0.5	$\sqrt{\frac{\varphi }{1-2{\left(1+\chi \right)}^{2}}}$	0<u<1	λ>0	$\beta <\frac{1}{\underset{j=1,\ldots ,N}{\max}\sum_{i=1}^{N}\left|a_{ij}\right|+d_{j}}$

## 3. Controller design

### 3.1. Event-triggered PPD update

Considering the data transmission process with output constrained (2), the following event-triggered pseudo partial derivative (PPD) updating law is devised:


$${\hat{\Gamma }}_{j}\left(k,t\right)={\hat{\Gamma }}_{j}\left(k-1,t\right)+$$

$$Q_{j}\left(k-1,t\right)\frac{\rho \Delta u_{j}\left(k-1,t\right)}{\vartheta +{\left|\Delta u_{j}\left(k-1,t\right)\right|}^{2}}\times$$

$$\left(\Delta z_{j}\left(k-1,t+1\right)-{\hat{\Gamma }}_{j}\left(k-1,t\right)\Delta u_{j}\left(k-1,t\right)\right)$$ (6)

$$Q_{j}\left(k-1,t\right)= \{\begin{array}{l} 1,\text{ triggering},\;t=t_{v}\\ 0,\text{ non-triggering, }t_{v-1}<t<t_{v} \end{array}$$ (7)

$t_{v}$ is the *v*th trigger moment, $t_{v-1}$ is the v−1 th trigger moment.

When $\left|\Delta u_{j}(k-1,t)\right|\leq \theta$, or $sign\left({\hat{\Gamma }}_{j}\left(k,t\right)\right)\neq sign\left({\hat{\Gamma }}_{j}\left(1,t\right)\right)$,


$${\hat{\Gamma }}_{j}\left(k,t\right)={\hat{\Gamma }}_{j}\left(k-1,t\right);$$ (8)

where ${\hat{\Gamma }}_{j}(k,t)$ is an estimate of the pseudo partial derivative $\Gamma_{j}(k,t)$, *ρ* is the step factor, and 0<ρ<1, ϑ>0 is a weighting factor to regulate excessive variations in the pseudo-skewed derivatives.

**Theorem 1** If assumption 3 holds, updating (6) ensures that $\Gamma_{j}(t)$ is bounded.

Proof:

#### 3.1.1. Trigger moments.

$Q_{j}\left(k-1,t\right)=1$, defining ${\bar{\Gamma }}_{j}\left(k,t\right)={\hat{\Gamma }}_{j}\left(k,t\right)-$$\Gamma_{j}\left(k,t\right)$, then, according to the event-triggered PPD update algorithm (6) could get:


$${\bar{\Gamma }}_{j}\left(k,t\right)=\left(1-\frac{\rho {\left|\Delta u_{j}\left(k-1,t\right)\right|}^{2}}{\delta +{\left|\Delta u_{j}\left(k-1,t\right)\right|}^{2}}\right)\times$$

$${\bar{\Gamma }}_{j}\left(k-1,t\right)-\Delta \Gamma_{j}\left(k,t\right)$$ (9)

owing to:

${\left|\Delta u_{j}\left(k-1,t\right)\right|}^{2}\neq 0$, 0<ρ<1, and δ>0 evidently ${\left|\Delta u_{j}\left(k-1,t\right)\right|}^{2}/\left(\delta +{\left|\Delta u_{j}\left(k-1,t\right)\right|}^{2}\right)<1$. So there must be a constant $q_{1}$ such that:


$$0<1-\frac{\rho {\left|\Delta u_{j}\left(k-1,t\right)\right|}^{2}}{\delta +{\left|\Delta u_{j}\left(k-1,t\right)\right|}^{2}}<q_{1}<1$$ (10)

The compact dynamic linearized model of the system shows that $\left|\Gamma_{j}(k,t)\right|\leq r$. And then from assumption 1 it follows that:


$$\left|\Delta \Gamma_{j}(k,t)\right|\leq \left|\Gamma_{j}(k,t)\right|\leq r$$ (11)

Therefore, from (9)–(11), it can be obtained:


$$\left|{\bar{\Gamma }}_{j}\left(k,t\right)\right|\leq q_{1}\left|{\bar{\Gamma }}_{j}\left(k-1,t\right)\right|+\left|\Delta \Gamma_{j}\left(k,t\right)\right|$$

$$\leq q_{1}^{k-1}\left|{\bar{\Gamma }}_{j}\left(1,t\right)\right|+\frac{r\left(1-q_{1}^{k-1}\right)}{1-q_{1}}$$ (12)

which can be further obtained as $\underset{k\to \infty }{\lim}\left|{\bar{\Gamma }}_{j}\left(1,t\right)\right|=$$r/(1-q_{1})$. It can be shown that ${\bar{\Gamma }}_{j}\left(1,t\right)$ is bounded, by lemma 1 it follows that $\Gamma_{j}(k,t)$ is bounded, so $\left|{\hat{\Gamma }}_{j}(k,t)\right|$ must be bounded. It can therefore be assumed that $\left|{\hat{\Gamma }}_{j}(k,t)\right|<\hat{r}$.

#### 3.1.2. Non-triggering moments.

$Q_{j}\left(k-1,t\right)=0$, from (6), ${\hat{\Gamma }}_{j}(k,t)=$${\hat{\Gamma }}_{j}(k-1,t)$, by lemma 1, $\Gamma_{j}(k,t)$ is bounded, so it follows that $\left|{\hat{\Gamma }}_{j}(k,t)\right|$ must be bounded.

This ends the proof.

### 3.2. Event-triggered communication mechanism design

Design the output observer of the system model as follows:


$${\hat{z}}_{j}\left(k,t+1\right)={\hat{z}}_{j}\left(k-1,t+1\right)+{\hat{\Gamma }}_{j}\left(k,t\right)\Delta u_{j}^{Ε}\left(k,t\right)+\chi \varepsilon_{ej}^{Ε}\left(k-1,t+1\right)$$ (13)

where ${\hat{z}}_{j}\left(k,t+1\right)$ is the output of the observer, $\Delta u_{j}^{Ε}\left(k,t\right)$ is an input to the system, $\varepsilon_{ej}^{Ε}\left(k-1,t+1\right)$ is the output estimation error, *χ* is the feedback gain of the observer.


$$\Delta u_{j}^{Ε}\left(k,t\right)=\Delta u_{j}\left(k,t_{v}\right)$$

$$t_{v}<t<t_{v+1}$$ (14)

where $t_{v+1}$ is the v+1 th trigger moment.


$$\varepsilon_{ej}^{Ε}\left(k-1,t+1\right)={\hat{z}}_{j}\left(k-1,t+1\right)-z_{j}^{Ε}(k-1,t+1)$$ (15)

$$z_{j}^{Ε}(k-1,t+1)=z_{j}(k-1,t_{v}+1)$$ (16)

where $t_{v}<t<t_{v+1}$.

From (14) and (16), we can see that, the input and output values of the observer are related to whether the system is triggered or not, when the system is triggered, the inputs and outputs of the observer are updated normally; when the system is not non-triggered, the inputs and outputs of the observer will remain as they were at the time of the last trigger.

The event triggering conditions for the agent *j* are defined as follows:


$$\eta \left(\left|\varepsilon_{j}\left(k,t\right)\right|\right)>\sqrt{\frac{u\left(1-4(1+\chi^{2})\right)}{4{\hat{r}}^{2}}}\left|\varepsilon_{ej}\left(k-1,t+1\right)\right|$$ (17)

where, $\varepsilon_{j}\left(k,t\right)$ is the output gain error of the observer, defining as:


$$\varepsilon_{j}\left(k,t\right)=\Delta u_{j}\left(k,t\right)-\Delta u_{j}^{Ε}(k,t)$$ (18)

$\varepsilon_{ej}\left(k,t+1\right)$ is the output estimation error of the observer, defining as:


$$\varepsilon_{ej}\left(k,t+1\right)={\hat{z}}_{j}\left(k,t+1\right)-z_{j}(k,t+1)$$ (19)

$\eta \left(\cdot \right)$ is a deadband controller function, defining as:


$$\eta \left(\left|\varepsilon_{j}\left(k,t\right)\right|\right)= \{\begin{array}{l} \left|\varepsilon_{j}\left(k,t\right)\right|,\left|\varepsilon_{ej}\left(k,t\right)\right|>\tau \\ 0,\left|\varepsilon_{ej}\left(k,t\right)\right|\leq \tau \end{array}$$ (20)

*τ* is a very small normal number, it will be analyzed in theorem 2. 0<u<1, $\hat{r}$ is PPD estimate of the last.

**Theorem 2** If the compact form dynamic linearized model of the system satisfies assumptions 1–3, and $\Gamma_{j}(k,t)$ are updated using the event-triggered PPD update (6) and (7), and the system satisfies event triggering condition (17), then consensus measurement error $\varepsilon_{ej}\left(k,t\right)$ is bounded.

**Proof:**

Substituting (13) into (19), and then from (3), (15), and (18), we can get:


$$\varepsilon_{ej}\left(k,t+1\right)=\left(1+\chi \right)\varepsilon_{ej}\left(k-1,t+1\right)+\chi E_{j}\left(k-1,t+1\right)-$$

$$\Delta z_{j}\left(k,t+1\right)+{\hat{\Gamma }}_{j}\left(k,t\right)\Delta u_{j}\left(k,t\right)$$

$$-{\hat{\Gamma }}_{j}\left(k,t\right)\varepsilon_{j}(k,t)$$ (21)

where $E_{j}\left(k-1,t+1\right)=z_{j}\left(k-1,t+1\right)-z_{j}^{Ε}(k-1,t+1)$. Owing to $\Delta z_{j}\left(k,t+1\right)=\Gamma_{j}(k,t)\Delta u_{j}\left(k,t\right)$, $0<\Gamma_{j}(k,t)<r$, and $\left|\Delta u_{j}\left(k,t\right)\right|<b$, thus it follows that $\Delta z_{j}\left(k,t\right)$ is bounded. From this, we can see, $E_{j}\left(k-1,t+1\right)$ is bounded, and there must be *α* constant such that $E_{j}\left(k-1,t+1\right)<\alpha$. Next, the boundedness of $\varepsilon_{ej}\left(k,t+1\right)$ is analyzed in terms of both triggering and non-triggering moments.

#### 3.2.1. Triggering moments.

When the system satisfies the event triggering conditions, from (14) and (16), we can obtain:

$z_{j}^{Ε}\left(k-1,t+1\right)=z_{j}\left(k-1,t_{v}+1\right)$, $\Delta u_{j}^{Ε}\left(k,t\right)=\Delta u_{j}\left(k,t\right)$, therefore, it is clear that $E_{j}\left(k-1,t+1\right)=0$, $\varepsilon_{j}\left(k,t\right)=0$, substituting them into (21) can obtain:


$$\varepsilon_{ej}\left(k,t\right)=\left(1+\chi \right)\varepsilon_{ej}\left(k-1,t+1\right)+$$

$${\hat{\Gamma }}_{j}\left(k,t\right)\Delta u_{j}\left(k,t\right)-\Delta z_{j}\left(k,t+1\right)$$ (22)

Define the Lyapunov function as follows:

$V_{j}\left(k,t+1\right)=\varepsilon_{ej}{\left(k,t+1\right)}^{2}$,

therefor


$$\Delta V_{j}\left(k,t+1\right)=V_{j}\left(k,t+1\right)-V_{j}\left(k-1,t+1\right)$$

$$=\varepsilon_{ej}{\left(k,t+1\right)}^{2}-\varepsilon_{ej}{\left(k-1,t+1\right)}^{2}$$ (23)

Substituting (22) into (23) can obtain:

$\Delta V_{j}\left(k,t+1\right)={\left(1+\chi \right)}^{2}\varepsilon_{ej}{\left(k-1,t+1\right)}^{2}+2\left(1+\chi \right)\varepsilon_{ej}\left(k-1,t+1\right)\left[{\hat{\Gamma }}_{j}\left(k,t\right)\Delta u_{j}\left(k,t\right)-\Delta z_{j}\left(k,t+1\right)\right]$$+{\left[{\hat{\Gamma }}_{j}\left(i,k\right)\Delta u_{j}\left(k,t\right)-\Delta z_{j}\left(k,t+1\right)\right]}^{2}-\varepsilon_{ej}{\left(k-1,t+1\right)}^{2}$,

once again

$2\left(1+\chi \right)\varepsilon_{ej}\left(k-1,t+1\right)\left[{\hat{\Gamma }}_{j}\left(k,t\right)\Delta u_{j}\left(k,t\right)-\Delta z_{j}\left(k,t+1\right)\right]\leq {\left[{\hat{\Gamma }}_{j}\left(k,t\right)\Delta u_{j}\left(k,t\right)-\Delta z_{j}\left(k,t+1\right)\right]}^{2}+$${\left(1+\chi \right)}^{2}\varepsilon_{ej}{\left(k-1,t+1\right)}^{2}$, consequently,


$$\Delta V_{j}\left(k,t+1\right)\leq -\left(1-2{\left(1+\chi \right)}^{2}\right)\varepsilon_{ej}{\left(k-1,t+1\right)}^{2}+2{\left({\hat{\Gamma }}_{j}\left(k,t\right)\Delta u_{j}\left(k,t\right)-\Delta z_{j}\left(k,t+1\right)\right)}^{2}$$ (24)

once again ${\bar{\Gamma }}_{j}\left(k,t\right)={\hat{\Gamma }}_{j}\left(k,t\right)-\Gamma_{j}\left(k,t\right)$, according to the compact form of the system dynamic linearized dynamic model (3) can be obtained as:


$$\Delta V_{j}\left(k,t+1\right)\leq -\left(1-2{\left(1+\chi \right)}^{2}\right)\varepsilon_{ej}{\left(k-1,t+1\right)}^{2}+2{\left[{\hat{\Gamma }}_{j}\left(k,t\right)-\Gamma_{j}\left(k,t\right)\right]}^{2}\Delta u_{j}{\left(k,t\right)}^{2}$$

$$\leq -\left(1-2{\left(1+\chi \right)}^{2}\right)\varepsilon_{ej}{\left(k-1,t+1\right)}^{2}+2{\bar{r}}^{2}b^{2}$$ (25)

enable $\varphi =2{\bar{r}}^{2}b^{2}$, $\bar{r}=\frac{r}{1-q_{1}}$. Consequently, it follows that when the following equation holds:


$$\left|\varepsilon_{ej}\left(k-1,t+1\right)\right|>\sqrt{\frac{\varphi }{1-2{\left(1+\chi \right)}^{2}}}=\tau$$ (26)

there is $\Delta V_{j}\left(k,t+1\right)<0$, it further follows that $\varepsilon_{ej}\left(k,t\right)$ is bounded.

#### 3.2.2. Non-triggering moments.

When the system is non-triggering moment, $\varepsilon_{j}\left(k,t\right)\neq 0$, $E_{j}\left(k-1,t+1\right)\neq 0$, substituting (21) into (23) can obtain:

$\Delta V_{j}\left(k,t+1\right)={\left(1+\chi \right)}^{2}\varepsilon_{ej}{\left(k-1,t+1\right)}^{2}+\chi^{2}E_{j}{\left(k-1,t+1\right)}^{2}+{\left[{\hat{\Gamma }}_{j}\left(k,t\right)\Delta u_{j}\left(k,t\right)-\Delta z_{j}\left(k,t+1\right)\right]}^{2}$

$+{\hat{\Gamma }}_{j}{\left(k,t\right)}^{2}\varepsilon_{j}{\left(k,t\right)}^{2}+2 [\left(1+\chi \right)\varepsilon_{ej}\left(k-1,t+1\right)\chi E_{j}\left(k-1,t+1\right)+\left(1+\chi \right)\varepsilon_{ej}\left(k-1,t+1\right)\times$

$\left({\hat{\Gamma }}_{j}\left(k,t\right)\Delta u_{j}\left(k,t\right)-\Delta z_{j}\left(k,t+1\right)\right)+\left(1+\chi \right)\varepsilon_{ej}\left(k-1,t+1\right){\hat{\Gamma }}_{j}\left(k,t\right)\varepsilon_{j}\left(k,t\right)+\chi E_{j}\left(k-1,t+1\right)\times$ $\left({\hat{\Gamma }}_{j}\left(k,t\right)\Delta u_{j}\left(k,t\right)-\Delta z_{j}\left(k,t+1\right)\right)+\chi E_{j}\left(k-1,t+1\right){\hat{\Gamma }}_{j}\left(k,t\right)\varepsilon_{j}\left(k,t\right)$

$\left.+\left({\hat{\Gamma }}_{j}\left(k,t\right)\Delta u_{j}\left(k,t\right)-\Delta z_{j}\left(k,t+1\right)\right){\hat{\Gamma }}_{j}\left(k,t\right)\varepsilon_{j}\left(k,t\right)\right]$,

according to the inequality $2xy\leq x^{2}+y^{2}$, the above equation can be obtained:

$\Delta V_{j}\left(k,t+1\right)\leq -\left(1-4{\left(1+\chi \right)}^{2}\right)\varepsilon_{ej}{\left(k-1,t+1\right)}^{2}+4\chi^{2}E_{j}{\left(i-1,k+1\right)}^{2}+4{\left[{\hat{\Gamma }}_{j}\left(k,t\right)\Delta u_{j}\left(k,t\right)-\Delta z_{j}\left(k,t+1\right)\right]}^{2}$ $+4{\hat{\Gamma }}_{j}{\left(k,t\right)}^{2}\varepsilon_{j}{\left(k,t\right)}^{2}$


$$\leq -\left(1-4{\left(1+\chi \right)}^{2}\right)\varepsilon_{ej}^{2}\left(k-1,t+1\right)+4\chi^{2}\alpha^{2}+4{\hat{r}}^{2}\varepsilon_{j}^{2}\left(k,t\right)+4{\bar{r}}^{2}b^{2}$$ (27)

where $\bar{r}=\frac{r}{1-q_{1}}$. Then according to (17) and (20), it can be obtained:


$$\Delta V_{j}\left(k,t+1\right)\leq -\left(1-u\right)\left(1-4{\left(1+\chi \right)}^{2}\right)\times$$

$$\varepsilon_{ej}^{2}\left(k-1,t+1\right)+\theta$$ (28)

where $\theta \geq 4\chi^{2}\alpha^{2}+4{\bar{r}}^{2}b^{2}$, 0<u<1. From (23) and (27), it can be obtained:


$$V_{j}\left(k,t+1\right)\leq {\left(1-\left(1-u\right)\left(1-4{\left(1+\chi \right)}^{2}\right)\right)}^{k-1}V_{j}\left(1,t+1\right)+\frac{\theta \left(1-{\left(1-\left(1-u\right)\left(1-4{\left(1+\chi \right)}^{2}\right)\right)}^{k-1}\right)}{\left(1-\left(1-u\right)\left(1-4{\left(1+\chi \right)}^{2}\right)\right)}$$ (29)

where if $0<1-\left(1-u\right)\left(1-4{\left(1+\chi \right)}^{2}\right)<1$, then we can obtain:


$$\underset{k\to \infty }{\lim}\left|V_{j}(i,k+1)\right|=\frac{\theta }{1-\left(1-\left(1-u\right)\left(1-4{\left(1+\chi \right)}^{2}\right)\right)}$$ (30)

From (28), we can see that, *χ* must satisfy −1.5<χ<−0.5 when there is $\Delta V_{j}\left(k,t+1\right)<0$, and according to (30), it follows that $V_{j}\left(k,t+1\right)$ converges, therefore, it can be shown that $\varepsilon_{ej}\left(k,t\right)$ is bounded.

This ends the proof.

**Remark 4** From (17), (20), and (26), when the system is triggered several times in a row or $\left|\varepsilon_{ej}\left(k,t\right)\right|<\tau$, deadband controller function value is 0, that would break event triggering condition (17), this causes the system to leave the event-triggering state, and the system stops triggering, therefore, the designed deadband controller can effectively avoid the occurrence of Zeno-like phenomenon.

### 3.3. Control protocol design

Design the event-triggering distributed control protocol as follows:


$$u_{j}\left(k,t\right)=u_{j}\left(k-1,t\right)+Q_{j}\left(k-1,t\right)\times$$

$$\frac{\beta {\hat{\Gamma }}_{j}(k,t)}{\lambda +{\left|{\hat{\Gamma }}_{j}(k,t)\right|}^{2}}\zeta_{j}\left(k-1,t+1\right)$$ (31)

where λ>0, *β* is the stability weight, whose exact range will be given in theorem 3.

**Remark 5** The consensus measurement error, equation (4), is determined from the topology and the output observer value of each agent. Through the event-triggering condition (17), the state update of the output observer (13) and the PPD estimation (6) is realized by controlling the $Q_{j}\left(k-1,t\right)$ of the 1 and 0 state switching, and further realizing the state update of the control protocol (31).

**Lemma 2** If $M\left(j\right)$ is a subrandom matrix varying along the iteration axis, and the diagonal elements are all positive, then using *M* to represent all possible subrandom matrices $M\left(j\right)$, one obtains:

$\left\|M\left(P\right)M\left(P-1\right)\cdots M\left(1\right)\right\|\leq \delta$,

where 0<δ<1, *P* can be arbitrarily selected from the $M\left(j\right)$.

**Theorem 3** If the system model satisfies assumptions 1–6, and the event triggering mechanism (17)–(20) can be utilized to realize the state update of the control protocol (31), then when $r^{2}/4<\lambda_{min}<\lambda ,\beta$ satisfies the following equation $\beta <\frac{1}{\underset{j=1,\ldots ,N}{\max}\sum_{i=1}^{N}\left|a_{ij}\right|+d_{j}}$, the estimation error a of multi-agent system can be made bounded, i.e., the tracking error of all the agents is bounded.

**Proof:** defining


$$r_{j}\left(k,t\right)= \{\begin{array}{l} \frac{y_{d}\left(t\right)-y_{0j}}{e_{j}(k,t)},e_{j}(k,t)<y_{d}\left(t\right)-y_{0j}\\ 1,y_{d}\left(t\right)-y_{0j}\leq e_{j}\left(k,t\right)\leq y_{d}\left(t\right)+y_{0j}\\ \frac{y_{d}\left(t\right)+y_{0j}}{e_{j}(k,t)},y_{d}\left(t\right)+y_{0j}<e_{j}\left(k,t\right) \end{array}$$ (32)

$$e_{j}^{A}\left(k,t\right)=r_{j}\left(k,t\right)e_{j}(k,t)$$ (33)

From the above equation and assumption 4, it is easy to obtain $0<r_{j}\left(k,t\right)\leq 1$. For simplicity of analysis, record $r\left(k,t\right)=diag\left\{r_{1}\left(k,t\right),r_{2}\left(k,t\right),\cdots ,r_{N}\left(k,t\right)\right\}$, according to ${\hat{e}}_{j}\left(k,t\right)=y_{d}\left(t\right)-{\hat{z}}_{j}\left(k,t\right),$${\hat{e}}_{j}^{A}\left(k,t\right)=r_{j}\left(k,t\right){\hat{e}}_{j}(k,t)$. The consensus measurement error (4) can be rewritten as follows:


$$\zeta_{j}\left(k,t\right)=\sum_{i\in N_{j}}a_{i,j}\left({\hat{e}}_{i}^{A}\left(k,t\right)-{\hat{e}}_{j}\left(k,t\right)\right)+$$

$$d_{j}\left({\hat{e}}_{j}^{A}\left(k,t\right)\right)$$ (34)

For the purpose of proof analysis, define the following set of vectors:

$\hat{z}\left(k,t\right)={\left[{\hat{z}}_{1}\left(k,t\right),{\hat{z}}_{2}\left(k,t\right),\cdots ,{\hat{z}}_{N}\left(k,t\right)\right]}^{T}$,

$u\left(k,t\right)={\left[u_{1}\left(k,t\right),u_{2}\left(k,t\right),\cdots ,u_{N}\left(k,t\right)\right]}^{T}$,

$\zeta \left(k,t\right)={\left[\zeta_{1}\left(k,t\right),\zeta_{2}\left(k,t\right),\cdots ,\zeta_{N}\left(k,t\right)\right]}^{T}$,

${\bar{y}}_{d}\left(k,t\right)={\left[y_{d}\left(k,t\right),y_{d}\left(k,t\right),\cdots ,y_{d}\left(k,t\right)\right]}^{T}$,

$\hat{e}\left(k,t\right)={\left[{\hat{e}}_{1}\left(k,t\right),{\hat{e}}_{2}\left(k,t\right),\cdots ,{\hat{e}}_{N}\left(k,t\right)\right]}^{T}$,

$\varepsilon_{e}\left(k,t\right)={\left[\varepsilon_{e1}\left(k,t\right),\varepsilon_{e2}\left(k,t\right),\cdots ,\varepsilon_{eN}\left(k,t\right)\right]}^{T}$.

Therefore, Equation (35) is expressed as a vector as follows:


$$\zeta \left(k,t\right)=((L+D)r\left(k,t\right)⊗I_{m})\hat{e}\left(k,t\right)$$ (35)

the convergence of the tracking error of the system is analyzed in terms of triggering and non-triggering moments.

#### 3.3.1. Non-triggering moments.

When the system is in non-triggering moments, $Q_{j}\left(k-1,t\right)=0$, the control input is equal to the input value at the last trigger, this leads to an increase in the estimation error of the output of the observer, further leading to an increase in system tracking error, therefore, the value of the deadband controller function is no longer 0, the event triggering condition is satisfied, and the system enters the event triggering state.

#### 3.3.2. Triggering moments.

When the system is at the triggering moments, $Q_{j}\left(k-1,t\right)=1$, according to ${\hat{e}}_{j}\left(k,t\right)=y_{d}\left(t\right)-$${\hat{z}}_{j}\left(k,t\right)$ can obtain:


$$\hat{e}\left(k,t+1\right)={\bar{y}}_{d}\left(k,t+1\right)-\hat{z}(k,t+1)$$ (36)

once again ${\bar{y}}_{d}\left(k,t+1\right)={\bar{y}}_{d}\left(k-1,t+1\right)$, which leads to


$$\hat{e}\left(k,t+1\right)=\left(I-\beta \psi \left(k,t\right)r\left(k-1,t+1\right)\right)\times$$

$$\hat{e}\left(k-1,t+1\right)-\chi \varepsilon_{e}(k-1,t+1)$$ (37)

where $\psi \left(k,t\right)=\Omega \left(k,t\right)\left(L+D\right)$, $\Omega \left(k,t\right)=diag\left\{\theta_{1},\theta_{2},\cdots \theta_{N}\right\}$, and there is $\theta_{j}$ satisfying the following equation


$$0<\theta_{j}=\frac{\Gamma_{j}(k,t){\hat{\Gamma }}_{j}(k,t)}{\lambda +{\left|{\hat{\Gamma }}_{j}(k,t)\right|}^{2}}<\frac{r}{2\sqrt{\lambda_{min}}}<1$$ (38)

Therefore, according to $0<r_{j}\left(k,t\right)\leq 1$ and the range of values of *β* in theorem 3, it is known that the row sum of the matrix $I-\beta \psi \left(k,t\right)r\left(k-1,t+1\right)$ must be less than 1, the matrix $I-\beta \psi \left(k,t\right)r\left(k-1,t+1\right)$ is then a subrandom matrix. Which can be further obtained as:


$$\left\|\hat{e}\left(k,t+1\right)\right\|\leq \left\|I-\beta \psi \left(k,t\right)r\left(k-1,t+1\right)\right\|\times$$

$$\left\|\hat{e}\left(k-1,t+1\right)\right\|+\left\|\chi \varepsilon_{e}\left(k-1,t+1\right)\right\|$$

$$\leq \left\|I-\beta \psi \left(k,t\right)r\left(k-1,t+1\right)\right\|\times$$

$$\left\|I-\beta \psi \left(k-1,t\right)r\left(k-2,t+1\right)\right\|\times \cdots$$

$$\times \left\|I-\beta \psi \left(2,t\right)r\left(1,t+1\right)\right\|\left\|\hat{e}\left(1,t+1\right)\right\|+\omega +\left\|I-\beta \psi \left(k,t\right)r\left(k-1,t+1\right)\right\|\omega +\cdots$$

$$+\left\|I-\beta \psi \left(k-1,t\right)r\left(k-2,t+1\right)\right\|\times \cdots$$

$$\times \left\|I-\beta \psi \left(3,t\right)r\left(2,t+1\right)\right\|\omega$$ (39)

From the above analysis, *χ* and $\varepsilon_{e}(k-1,t+1)$ are bounded, so there must be a constant *ω* such that $\left\|\chi \varepsilon_{e}(k-1,t+1)\right\|<\omega$, 0<ω<1.

From lemma 2 and (37), we can obtain:


$$\left\|\hat{e}\left(k,t+1\right)\right\|\leq \left(\delta^{\frac{k-2}{P}}+\delta^{\frac{k-3}{P}}+\cdots +\delta^{\frac{0}{P}}\right)+$$

$$\delta^{\frac{k-1}{P}}\left\|\hat{e}\left(1,t+1\right)\right\|$$

where defining $O\left(k\right)=\delta^{\frac{kP}{P}}+\delta^{\frac{kP+1}{P}}+\cdots +\delta^{\frac{\left(k+1\right)P-1}{P}}$, therefore, there is $O\left(k\right)=P\delta^{k}$, so it follows that


$$\underset{k\to \infty }{\lim}\left\|\hat{e}\left(k,t+1\right)\right\|$$

$$=\left(O\left(k\right)+O\left(k-1\right)+\cdots +O\left(0\right)\right)\omega$$

$$=\frac{P}{1-\delta }\omega$$

The above analysis shows that, $0<\frac{P}{1-\delta }\omega <1$ i.e. tracking estimation error is bounded. It follows that the tracking error $e_{j}\left(k,t\right)$ is bounded.

This ends the proof.

**Remark 6** From $\underset{k\to \infty }{\lim}\left\|\hat{e}\left(k,t+1\right)\right\|=\frac{P}{1-\delta }\omega$, it can be seen that the upper term of the tracking error is affected by the parameters b, r and *χ*. *χ* is the feedback gain of the observer, *b* and *r* respectively are bounded on the system inputs and pseudo-partial derivatives, when the system model is determined, b, r is basically unchanged, and the tracking error can be changed by changing the value of *χ*. The number of system triggers can be further adjusted to affect the convergence speed of the system.

## 4. Simulation results and discussion

In this section, the effectiveness of the proposed scheme is verified by a discrete-time nonlinear single-input single-output multi-agent system and a discrete-time nonlinear multi-input and multi-output system, the system consists of 1 leader and 5 followers. The simulation environment used in this paper: windows 10 system, x64 processor, MATLAB R2020a version.

**Fig 1:**
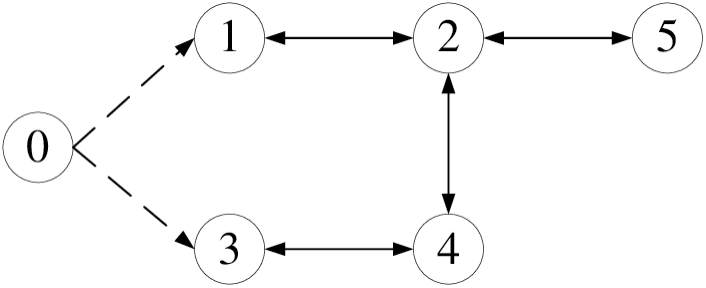
Communication topology.

**Example 1.** The effectiveness of the proposed scheme is verified by a discrete-time nonlinear single-input single-output multi-agent system, it is modeled as follows:


$$\{\begin{array}{l} \begin{array}{l} x_{k,j}\left(t+1\right)=\left[\begin{array}{c} -1\\ 0.7 \end{array}\right]u_{k,j}\left(t\right)+\left[\begin{array}{c} cos\left(0.67x_{k,j1}\left(t\right)+1.2x_{k,j2}\left(t\right)\right)\\ -1.2sin\left(0.8x_{k,j1}\left(t\right)+1.1x_{k,j2}\left(t\right)\right) \end{array}\right] \end{array}\\ y_{k,j}\left(t\right)=\left[\begin{array}{cc} 1 & 2 \end{array}\right]x_{k,j}(t) \end{array}$$

where $t\in \left[0,1,\ldots ,50\right]$ is the time interval, j∈1,2,…,5 denotes the *j*th agent, $k\in \left[0,1,\ldots ,500\right]$. The desired trajectory $y_{d}\left(t\right)=1.1sin\left(0.27t\right)+$cos(0.1πt) is given, the communication topology of this paper is shown in Fig 1.

As you can see from the communication topology diagram, the agents 1 and 3 have direct access to the leader’s information, and the agents 2, 4, and 5 cannot access the leader’s information, therefore, $D=diag\left\{1,0,1,0,0\right\}$. The Laplace matrix of Fig 1 is:


$$L=\left[\begin{array}{ccccc} 1 & -1 & 0 & 0 & 0\\ -1 & 3 & 0 & -1 & -1\\ 0 & 0 & 1 & -1 & 0\\ 0 & -1 & -1 & 2 & 0\\ 0 & -1 & 0 & 0 & 1 \end{array}\right]$$

The output limitation thresholds for each agent in the system are set to be respectively $\left[y_{01}y_{02}y_{03}y_{04}y_{05}\right]=\left[\begin{array}{lllll} 1.41 & 1.5 & 1.42 & 1.4 & 1.41 \end{array}\right]$. The state of each agent at the initial moment in the simulation is given separately:


$$x_{k,1}\left(0\right)={\left[\begin{array}{cc} 0 & 0.5 \end{array}\right]}^{T}$$

$$x_{k,2}\left(0\right)={\left[\begin{array}{cc} -0.2 & 0.6 \end{array}\right]}^{T}$$

$$x_{k,3}\left(0\right)={\left[\begin{array}{cc} -0.2 & 0.6 \end{array}\right]}^{T}$$

$$x_{k,4}\left(0\right)={\left[\begin{array}{cc} -0.2 & 0.6 \end{array}\right]}^{T}$$

$$x_{k,5}\left(0\right)={\left[\begin{array}{cc} -0.2 & 0.6 \end{array}\right]}^{T}$$

**Fig 2:**
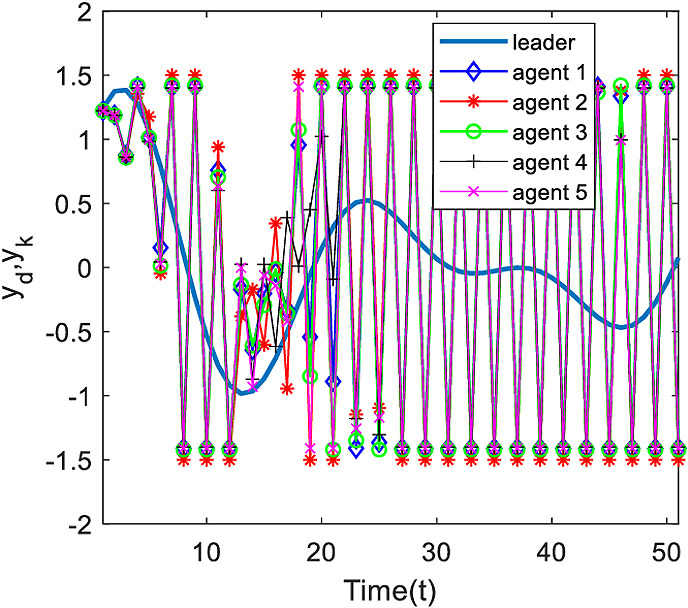
Output of each agent at time k=20.

The initial value is set as $u_{j}\left(1,t\right)=0$, ${\hat{\Gamma }}_{j}\left(1,t\right)=0.5$, the parameters are set to λ=0.1, ρ=0.3, χ=−1.4, u=1, ϑ=4, θ=0.0001, j=1,2,3,4,5. Since the maximum value of the diagonal elements in *L* +D is 3, set β=0.3, the experimental results are analyzed as follows:

Fig 2 gives the desired trajectory and the output of each agent in the 20th iteration, it can be found from the figure that, five agents have output constrained phenomenon under the event triggering mechanism, the output constrained restriction has a significant impact on the system.

**Fig 3:**
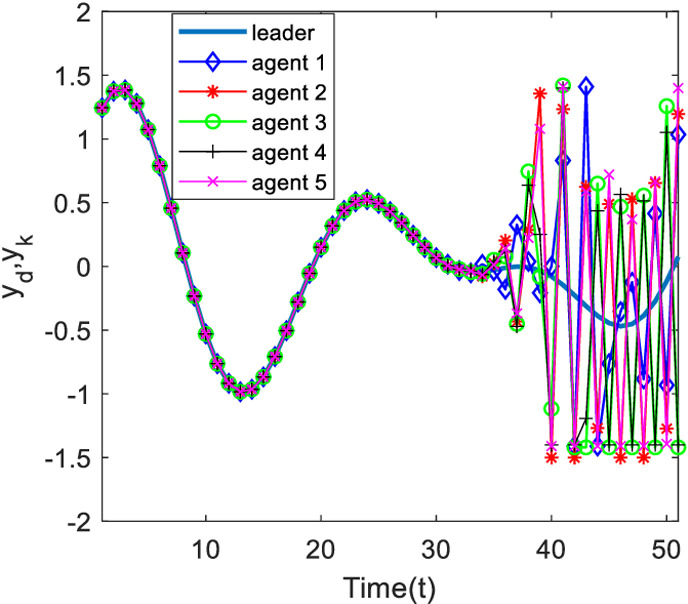
Output of each agent at time k=200.

**Fig 4:**
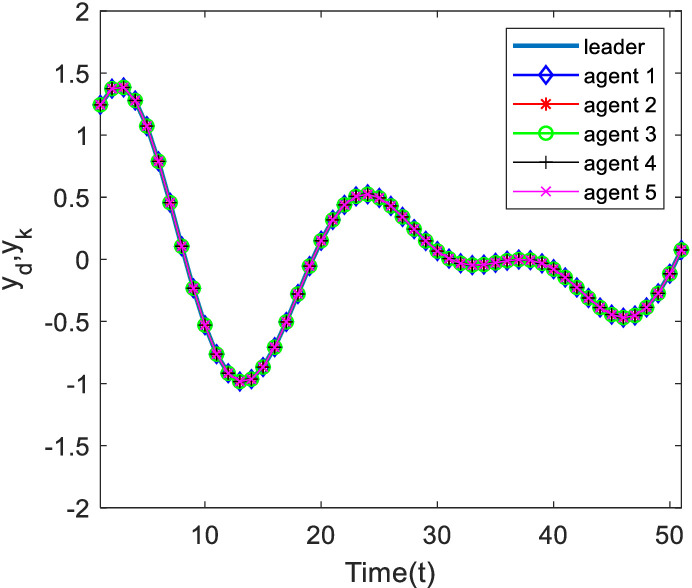
Output of each agent at time k=320.

Figs 3 and 4 show the outputs of the system at 200 and 500 iterations. Analyzing Fig 3, at 200 iterations, it can be seen from the figure that, under the control algorithm of this paper, the system output gradually approaches the desired output through the continuous correction of the system input by the measurement consensus error, and then the system tracking error gradually decreases, and the phenomenon of the system output limitation is weakened, but there is still a large error with the desired trajectory; with the increase of the number of iterations, at the 500th iteration, the output of each agent is completely tracking on the desired trajectory in the finite time interval.

**Fig 5:**
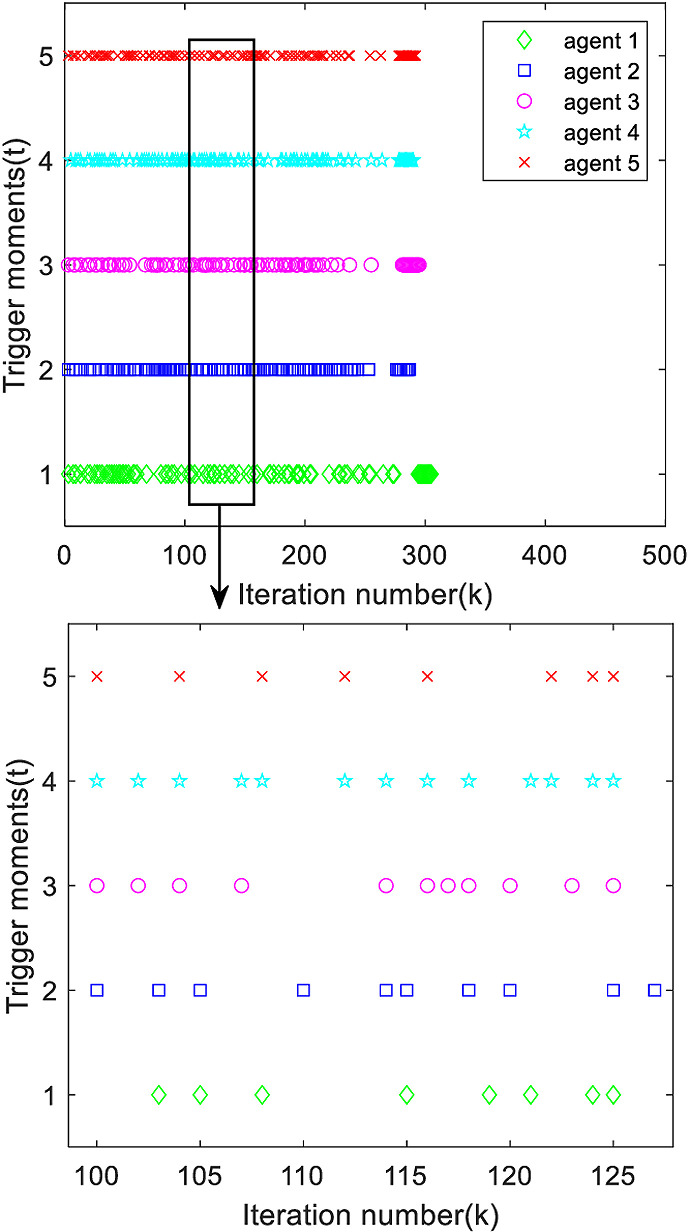
Trigger moments for each agent.

Fig 5 gives the triggering moments of each agent, it can be seen from the figure that the triggering moments of each agent are intermittent, so it is effectively verified that the designed deadband controller avoids Zeno behavior very well.

**Fig 6:**
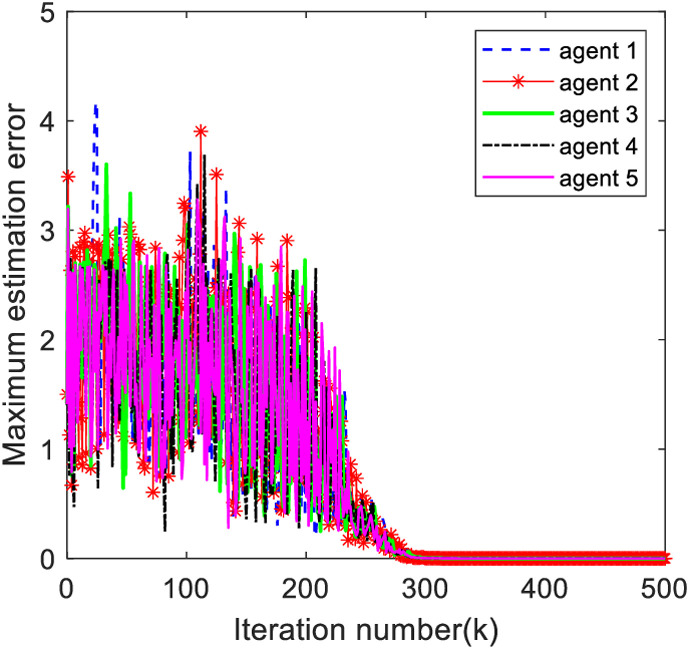
Maxmum output estimation error of the observer.

Fig 6 shows the maximum output estimation error of the system, from Fig 6, the maximum output estimation error is 0 at about 300 iterations, indicating that the output observer designed in this paper can effectively estimate the observer output.

Define the event trigger rate to reflect the number of communication requests from different agents in the direction of the iteration axis, $\eta_{j}=\left(c_{j}/N\right)\times 100\%$, where $c_{j}$ denotes the number of event-triggering communications of agent *j* during the iteration process, *N* denotes the number of iterations of the system. The event triggering rate of each agent is shown in Table 2.

**Table 2:** Trigger rate of events by agents.

Agent	1	2	3	4	5
Trigger rate	16.8%	19.6%	16.2%	18.8%	18.4%
Average trigger rate	17.96%				

**Fig 7:**
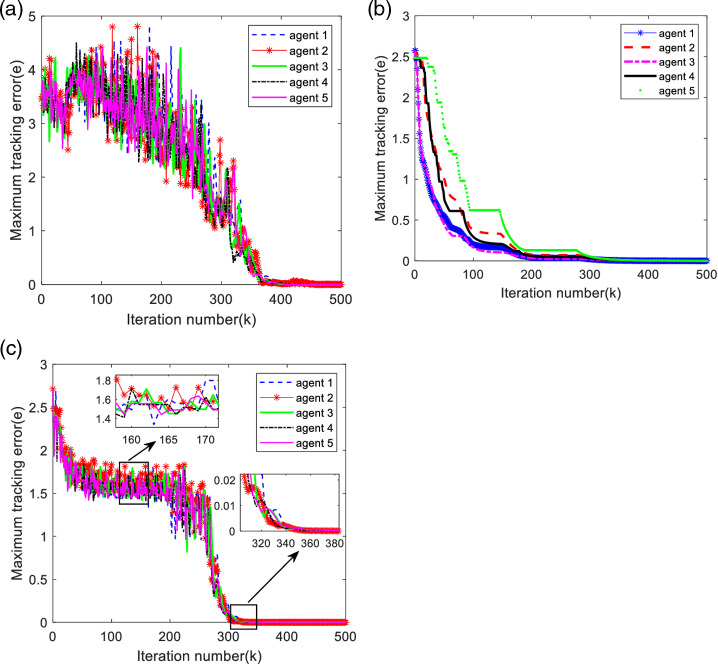
Comparison of maximum tracking error along the iteration axis. (a) No output constrained and event trigger. (b) Under output constrained and No event trigger. (c) Under output constrained and Event trigger.

**Fig 8:**
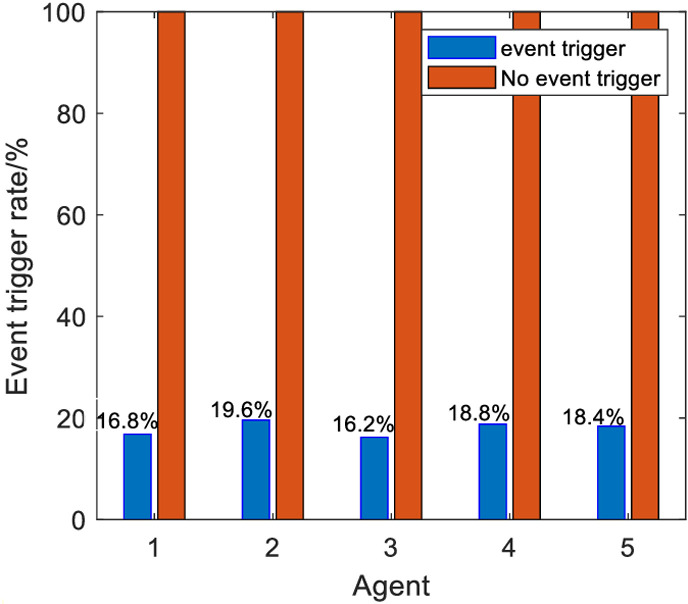
Comparison of event trigger rates.

In order to measure the impact of the algorithms of this paper on the effectiveness of control, the literatures [29,30] algorithms are compared with this paper’s algorithm. Fig 7a shows the maximum tracking error curve of the algorithm in literature [30], Fig 7b shows the maximum tracking error curve of the algorithm in literature [29], and Fig 7c shows the maximum tracking error curve of the algorithm in this paper. Comparing Fig 7a and 7c, it can be found that the control algorithm in this paper utilizes a small number of iterations to make the maximum tracking error of the system converge to 0 in a finite period of time, which indicates that the control algorithm in this paper can effectively solve the consistency tracking problem of the multi-intelligence system under the output constraints, and has good robustness. Comparing Fig 7b and 7c, it can be found that the algorithm in this paper has similar control effect with the algorithm in the literature [29], but from Fig 8, the algorithm of this paper has a low triggering rate, which is better than the iterative learning control algorithm with time-driven mechanism in terms of saving space resources and reducing energy loss. Therefore, the control algorithm proposed in this paper not only has good convergence performance, but also can better save space resources and energy loss.

**Fig 9:**
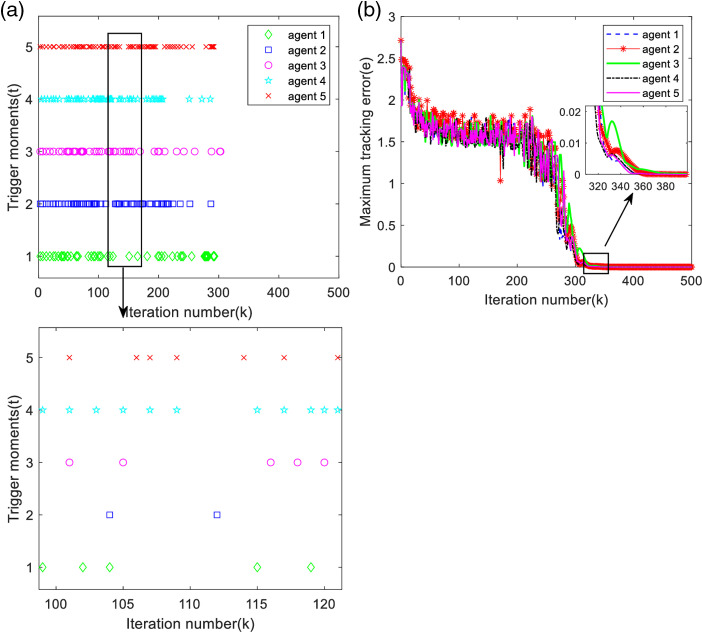
Maximum tracking error and trigger moment for each agent (χ=−1.2). (a) Trigger moments for each agent. (b) Maximum tracking error along the iteration axis.

**Fig 10:**
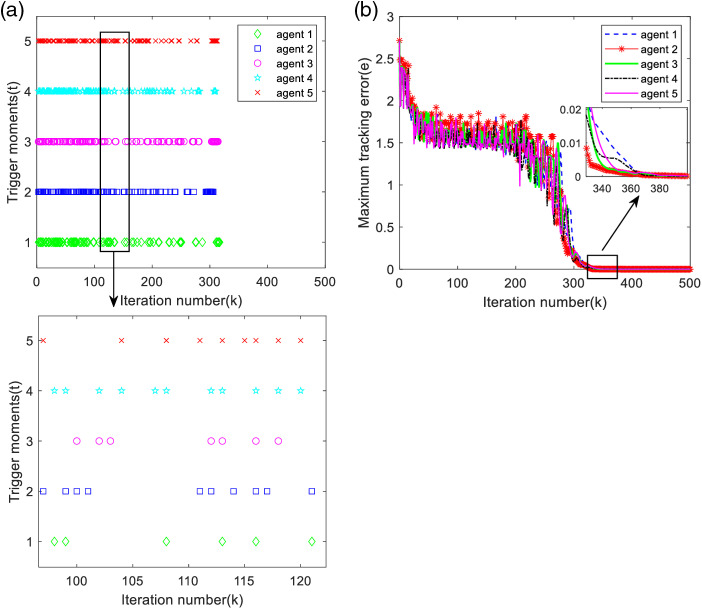
Maximum tracking error and trigger moment for each agent (χ=−0.6). (a) Trigger moments for each agent. (b) Maximum tracking error along the iteration axis.

From note 5, it can be found that the output tracking error of each agent is related to the value of *χ*. Fig 9 shows the maximum tracking error curve and trigger moment of each agent at time χ=−1.2, and Fig 10 shows the maximum tracking error curve and trigger moment of each agent at time χ=−0.6.

According to Figs 9a and 10a, it can be found that the triggering moments of each agent are intermittent, so the designed deadband controller can avoid the Zeno phenomenon. As analyzed in Table 3, the number of triggers becomes more in χ=−0.6, and the maximum tracking error converges slower; the number of triggers becomes less in χ=−1.2, but it needs more iteration number to arrive the bounded stability, i.e., the system takes longer time to reach stabilization, as observed in Figs 9b and 10b, and it can be seen that the value of *χ* affects the system performance.

**Table 3:** Trigger counts of each agent and average trigger counts of all agents (*χ* takes different values).

	χ=−1.4	χ=−1.2	χ=−0.6
Agent1	84	50	89
Agent2	98	63	98
Agent3	87	46	100
Agent4	94	56	103
Agent5	92	68	97
Average number of triggers	91	56.6	97.4

**Example 2.** The effectiveness of the proposed scheme is verified by a discrete-time nonlinear multi-input and multi-output system, the system is modeled as follows:


$$\{\begin{array}{l} x_{k,j}\left(t+1\right)=\left[\begin{array}{c} -x_{k,j1}\left(t\right)\\ \cos\left(x_{k,j1}\left(t\right)x_{k,j3}\left(t\right)\right)\\ -x_{k,j2}\left(t\right)-x_{k,j3}\left(t\right) \end{array}\right]+\left[\begin{array}{c} u_{k,j1}\left(t\right)\\ u_{k,j2}\left(t\right)\\ \sin\left(x_{k,j1}\left(t\right)u_{k,j2}\left(t\right)\right) \end{array}\right]\\ y_{k,j}\left(t\right)=\left[\begin{array}{c} x_{k,j1}\left(t\right)\\ x_{k,j2}\left(t\right) \end{array}\right] \end{array}$$

where $t\in \left[0,1,\ldots ,50\right]$ is the time interval, j∈1,2,…,5 denotes the jth agent, $k\in \left[0,1,\ldots ,500\right]$. The desired trajectory $y_{d1}=12t^{2}\left(1-t\right)$, $y_{d2}=\cos\left(\pi t\right)$ is given. The state of each agent at the initial moment in the simulation $x_{k,j}\left(0\right)={\left[\begin{array}{ccc} 0 & 1 & 0 \end{array}\right]}^{T}$ is given. The output limitation thresholds for each agent in the system are set to be $y_{j1}=2$, $y_{j2}=1.5$. The communication topology is the same as Example 1.

**Fig 11:**
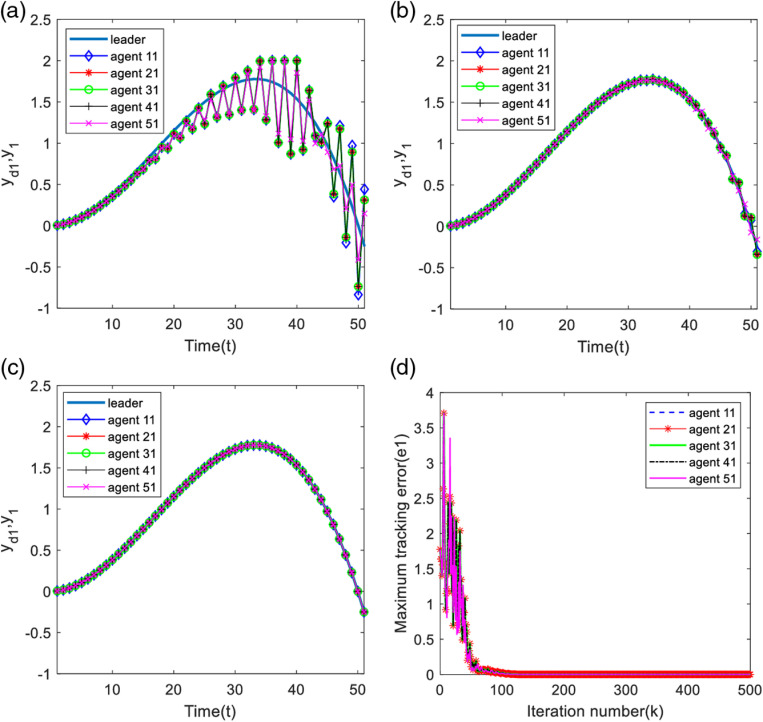
First output and maximum tracking error for each agent. (a) Output of each agent at time k=30. (b) Output of each agent at time k=60. (c) Output of each agent at time k=500. (d) Maximum tracking error along the iteration axis.

**Fig 12:**
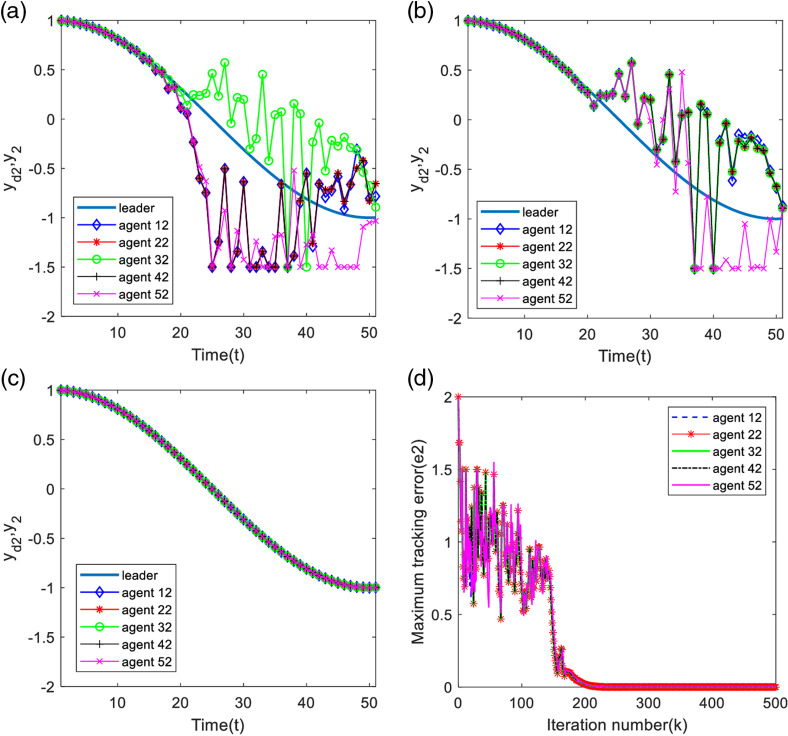
Second output and maximum tracking error for each agent. (a) Output of each agent at time k=30. (b) Output of each agent at time k=60. (c) Output of each agent at time k=500. (d) Maximum tracking error along the iteration axis.

The initial value is set as $u_{j}\left(1,t\right)={\left[\begin{array}{cc} 0 & 0 \end{array}\right]}^{T}$, ${\hat{\Gamma }}_{j}\left(1,t\right)=0.2$. The parameters are set to λ=0.7, ρ=0.3, χ=−0.9, u=1, ϑ=4, θ=0.0001, j=1,2,3,4,5. Since the maximum value of the diagonal elements in *L*+D is 3, set β=0.3, the experimental results are analyzed as follows:

Analyzing Figs 11a–d and 12a–d, in 30 iterations, the two outputs of the system have obvious output constrained phenomenon, with the increase of the number of iterations, in 60 iterations, the constrained phenomenon of the first output of the system disappeared, and the second output of the system is still constrained phenomenon. The two outputs of the system are completely tracking the desired trajectory when iteration is 500 times.

From Figs 11d and 12d, the first maximum tracking error is 0 at about 100 iterations, which indicates that the first output of the system has completely tracked the desired trajectory; the second maximum tracking error is 0 at about 200 iterations, which indicates that the second output of the system has completely tracked the desired trajectory.

**Fig 13:**
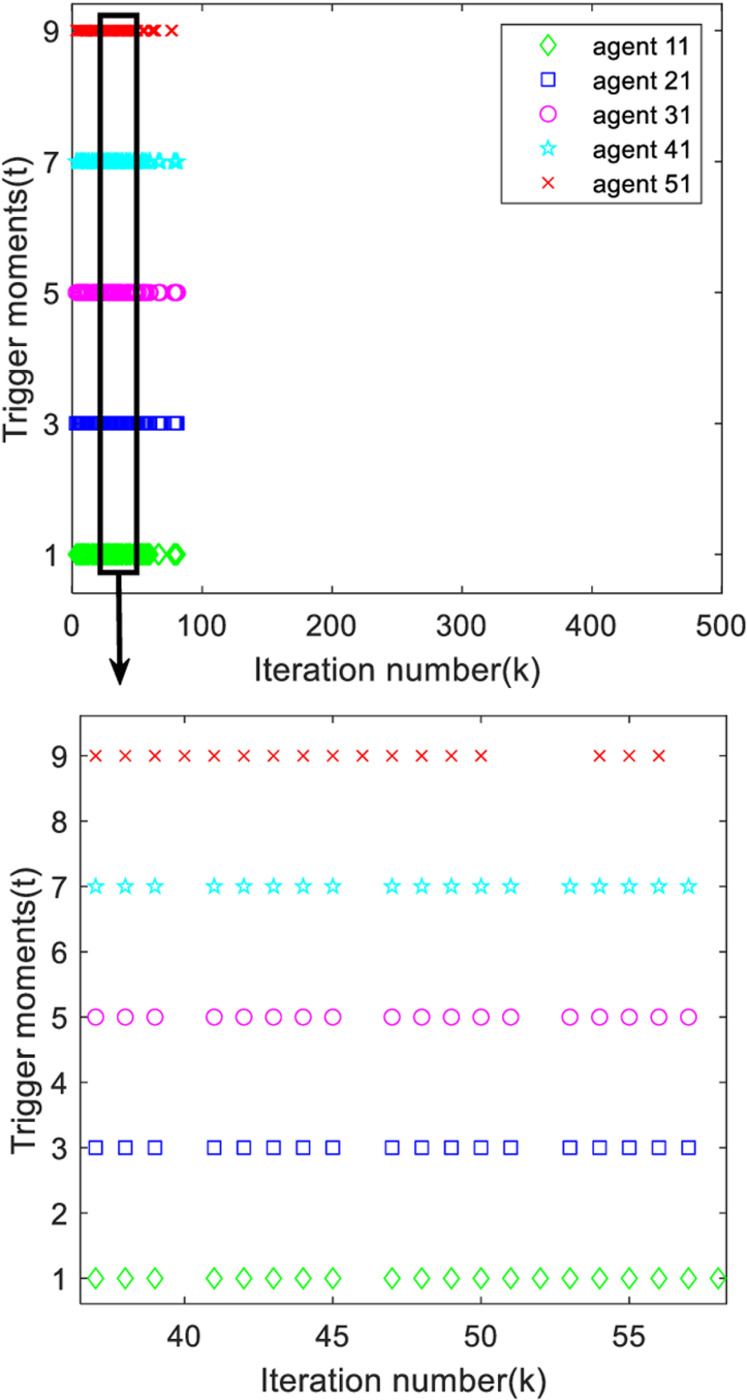
The first output trigger moment of each agent.

**Fig 14:**
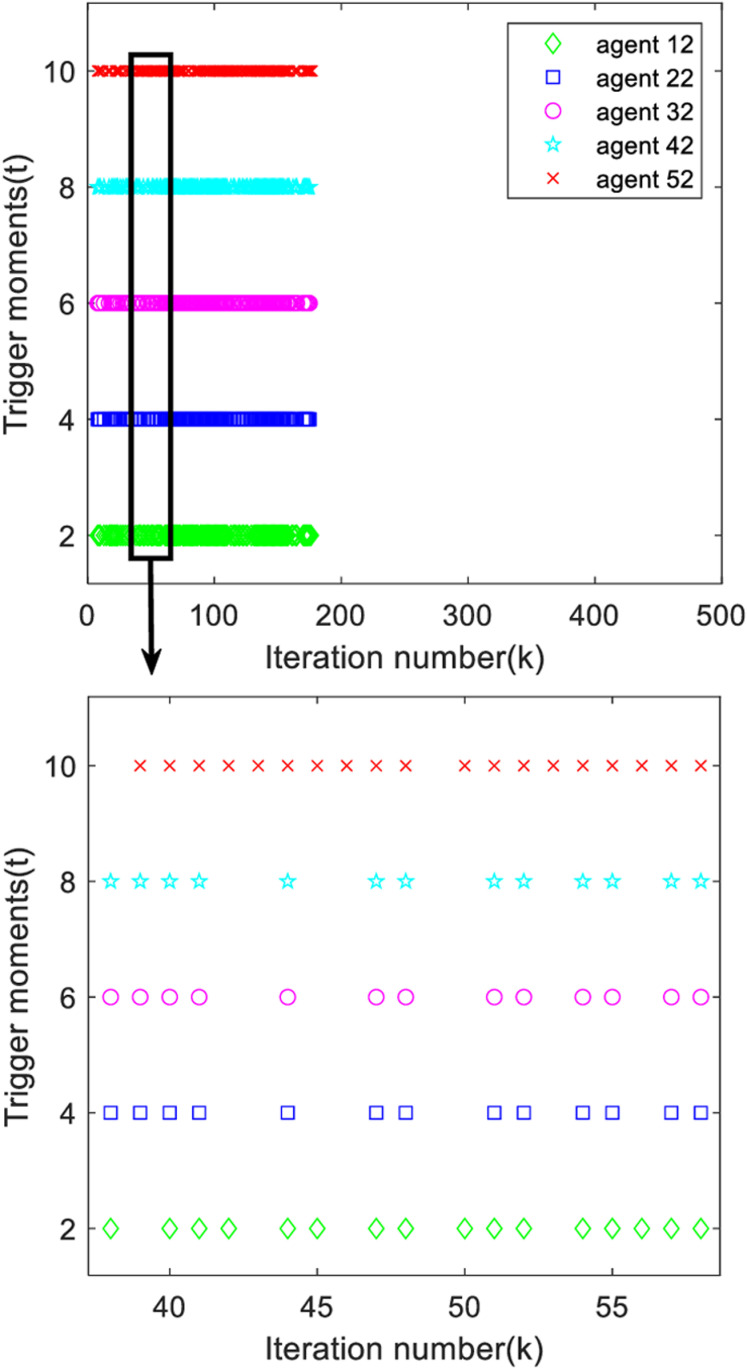
The second output trigger moment of each agent.

Analyzing Figs 13 and 14, it can be found that the system trigger moments are intermittent in a certain period time, proving that the designed deadband controller can effectively avoid the occurrence of Zeno phenomenon.

From the simulation results of Example 1 and Example 2, it can be found that the control algorithm in this paper can not only solve the consensus problem of single-input-single-output multi-agent systems, but also solve the consensus problem of multiple-input-multiple-output-multi-agent systems under the constraint of system output.

## 5. Conclusion

In this paper, an event-triggered distributed iterative learning control algorithm is proposed for the consistency problem of output constrained nonlinear multi-agent systems, and the convergence of the control algorithm is proved by using the Lyapunov function. The algorithm is simple in structure, has few parameters, does not need the model information of the controlled object, and can make the output constrained multi-agent systems track the desired trajectory consistently and completely in a finite time interval without the need of real-time communication. The designed output observer solves the problem that the measured values are not easy to obtain under the output constrained. The event-triggered mechanism effectively saves the system space and reduces the energy loss of the computer, and the designed deadband controller effectively avoids the Zeno behavior. Finally, it is verified that the control algorithm in this paper can also solve the consensus problem of multi-input multi-output multi-agent systems.

This paper mainly addresses the problem of consistent tracking and space saving for nonlinear systems under output constrained, and the event-triggered iterative learning control algorithm used is proposed without considering the noise interference, which may increase the tracking error of the system and the number of iterations. How to further reduce the number of iterations and improve the convergence speed of the system under noise interference will be one of the future research directions of this paper.

## Supporting information

S1 Data Simulation data for the figures and tables. (ZIP)
