# Supplementary figures and images for: Event-triggered iterative learning control for output constrained multi-agent systems
Source: PLoS One. 2025 Mar 21;20(3):e0315209. doi: 10.1371/journal.pone.0315209 (PMC11927920; doi:10.1371/journal.pone.0315209)

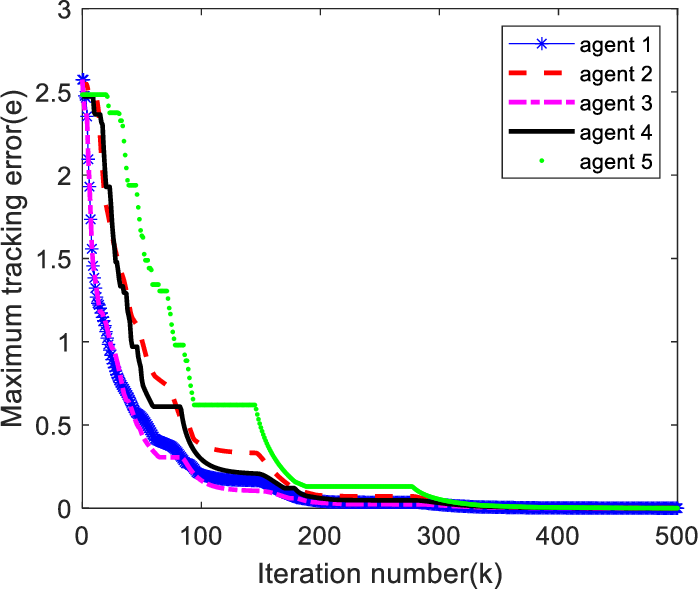

Supplement: S1 Data — (ZIP) [file pone.0315209.s001.zip › S1 data/Figures/Fig 7(b).tif]

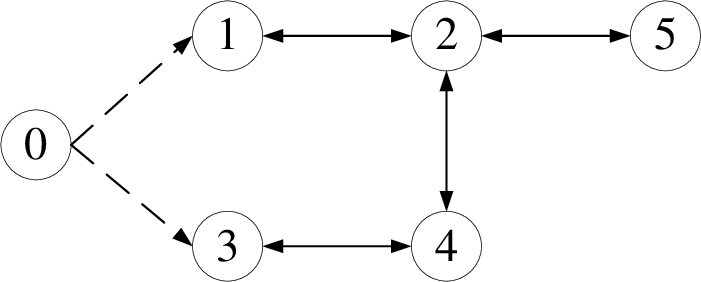

Supplement: S1 Data — (ZIP) [file pone.0315209.s001.zip › S1 data/Figures/Fig 1.tif]

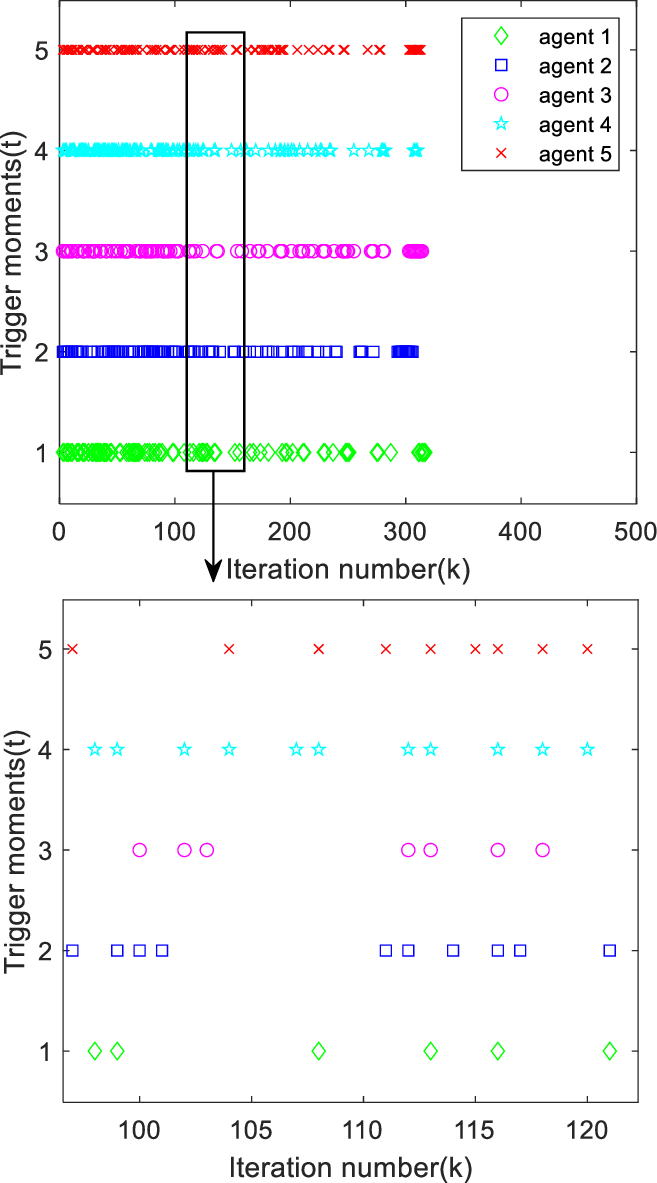

Supplement: S1 Data — (ZIP) [file pone.0315209.s001.zip › S1 data/Figures/Fig 10(a).tif]

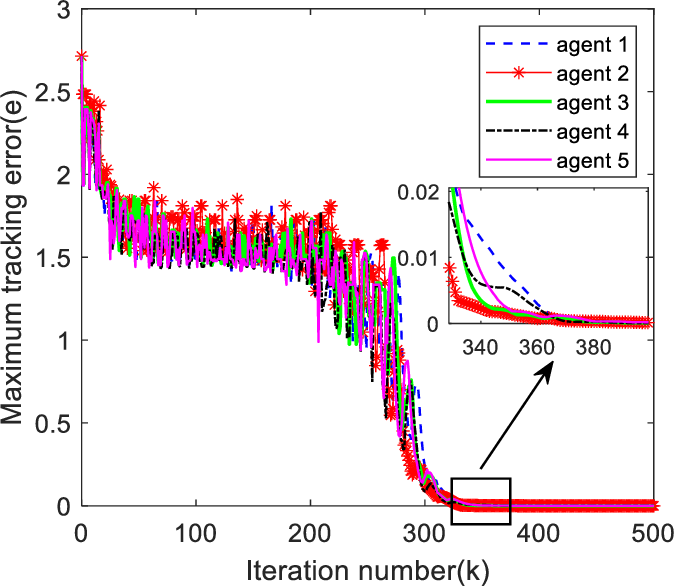

Supplement: S1 Data — (ZIP) [file pone.0315209.s001.zip › S1 data/Figures/Fig 10(b).tif]

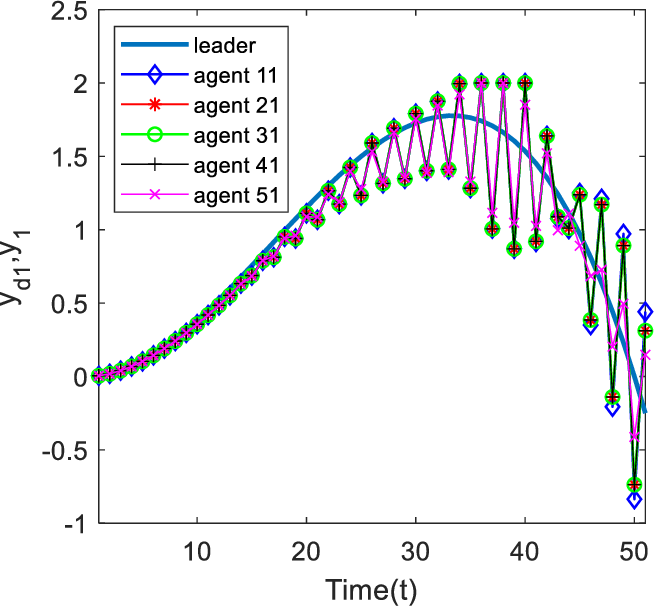

Supplement: S1 Data — (ZIP) [file pone.0315209.s001.zip › S1 data/Figures/Fig 11(a).tif]

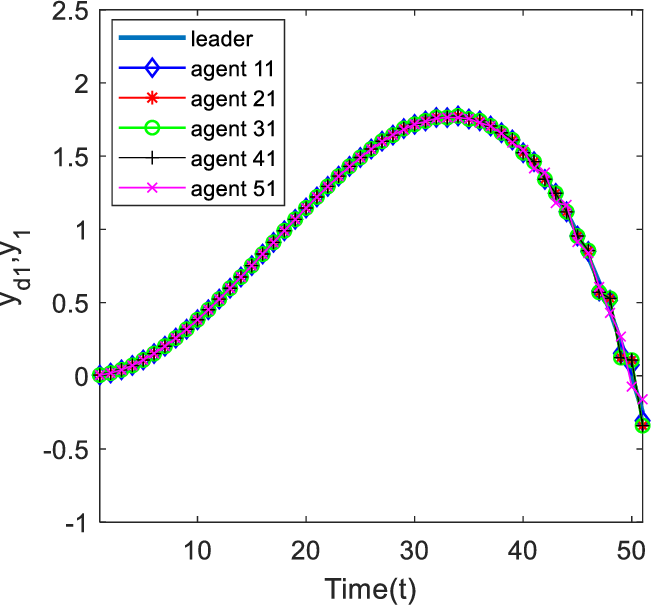

Supplement: S1 Data — (ZIP) [file pone.0315209.s001.zip › S1 data/Figures/Fig 11(b).tif]

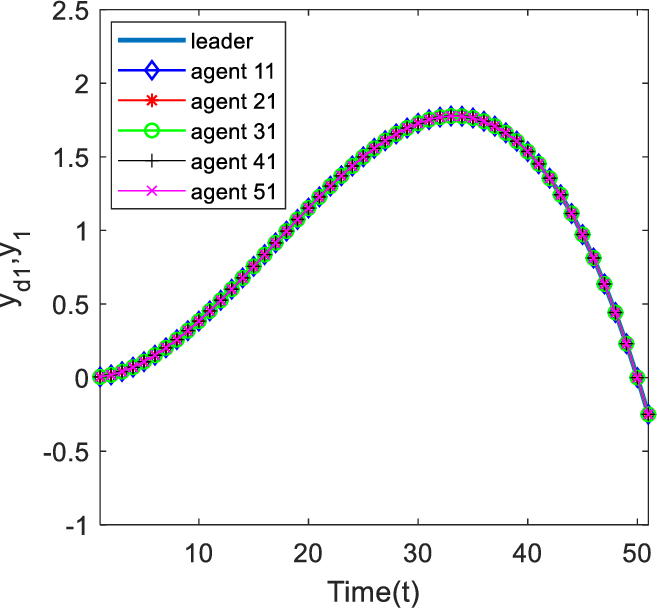

Supplement: S1 Data — (ZIP) [file pone.0315209.s001.zip › S1 data/Figures/Fig 11(c).tif]

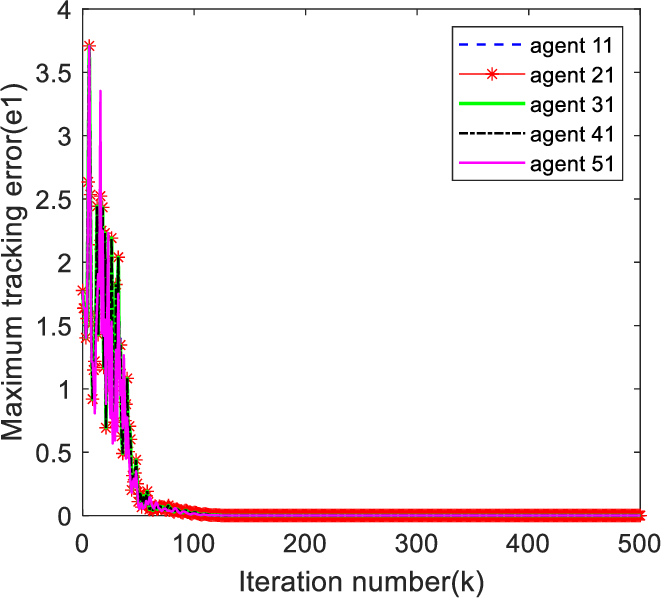

Supplement: S1 Data — (ZIP) [file pone.0315209.s001.zip › S1 data/Figures/Fig 11(d).tif]

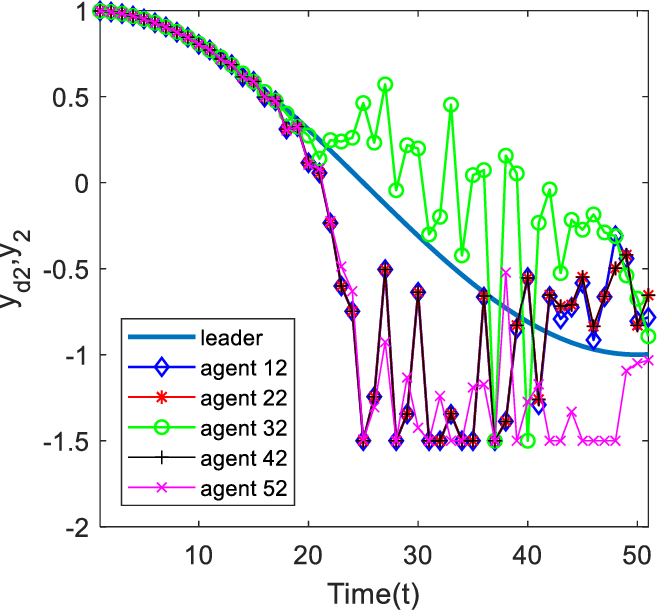

Supplement: S1 Data — (ZIP) [file pone.0315209.s001.zip › S1 data/Figures/Fig 12(a).tif]

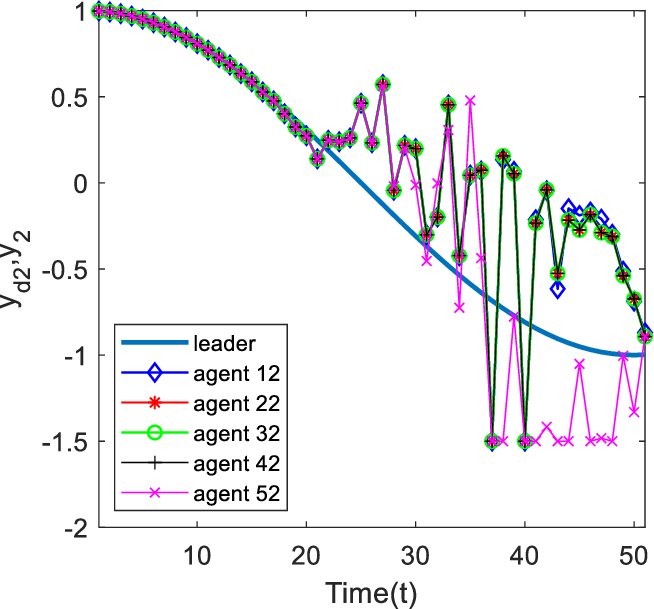

Supplement: S1 Data — (ZIP) [file pone.0315209.s001.zip › S1 data/Figures/Fig 12(b).tif]

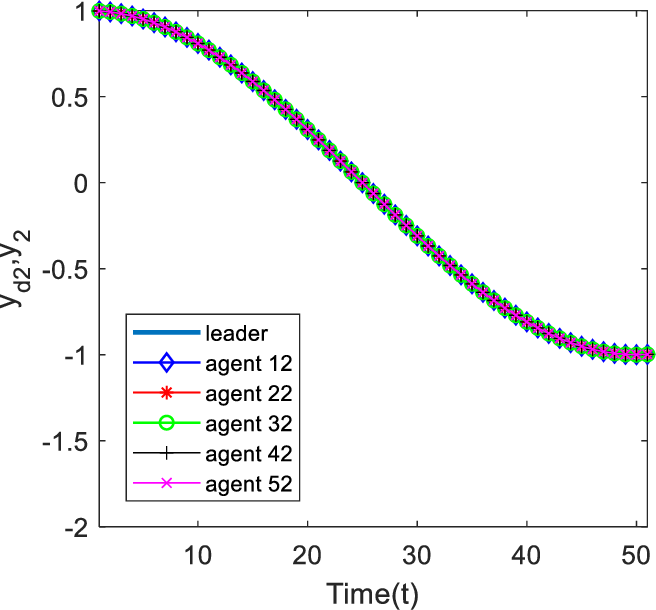

Supplement: S1 Data — (ZIP) [file pone.0315209.s001.zip › S1 data/Figures/Fig 12(c).tif]

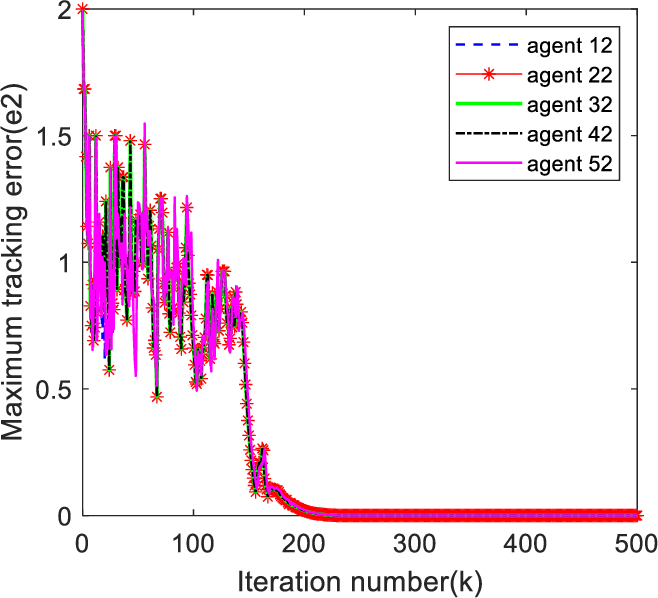

Supplement: S1 Data — (ZIP) [file pone.0315209.s001.zip › S1 data/Figures/Fig 12(d).tif]

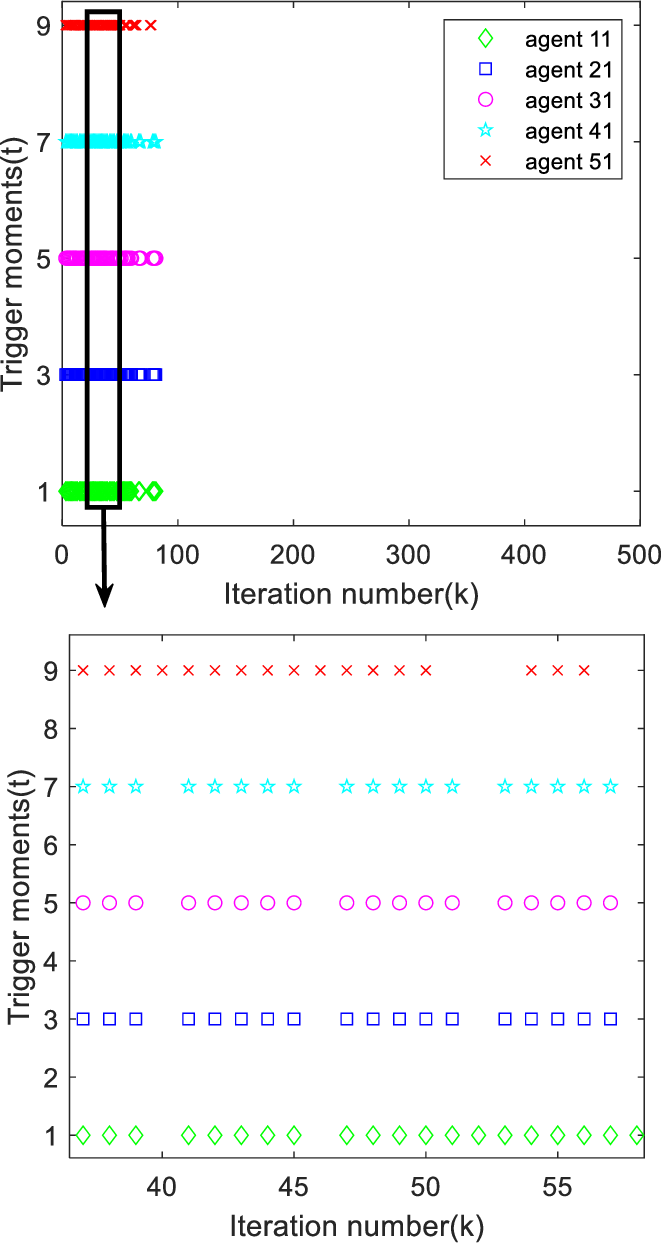

Supplement: S1 Data — (ZIP) [file pone.0315209.s001.zip › S1 data/Figures/Fig 13.tif]

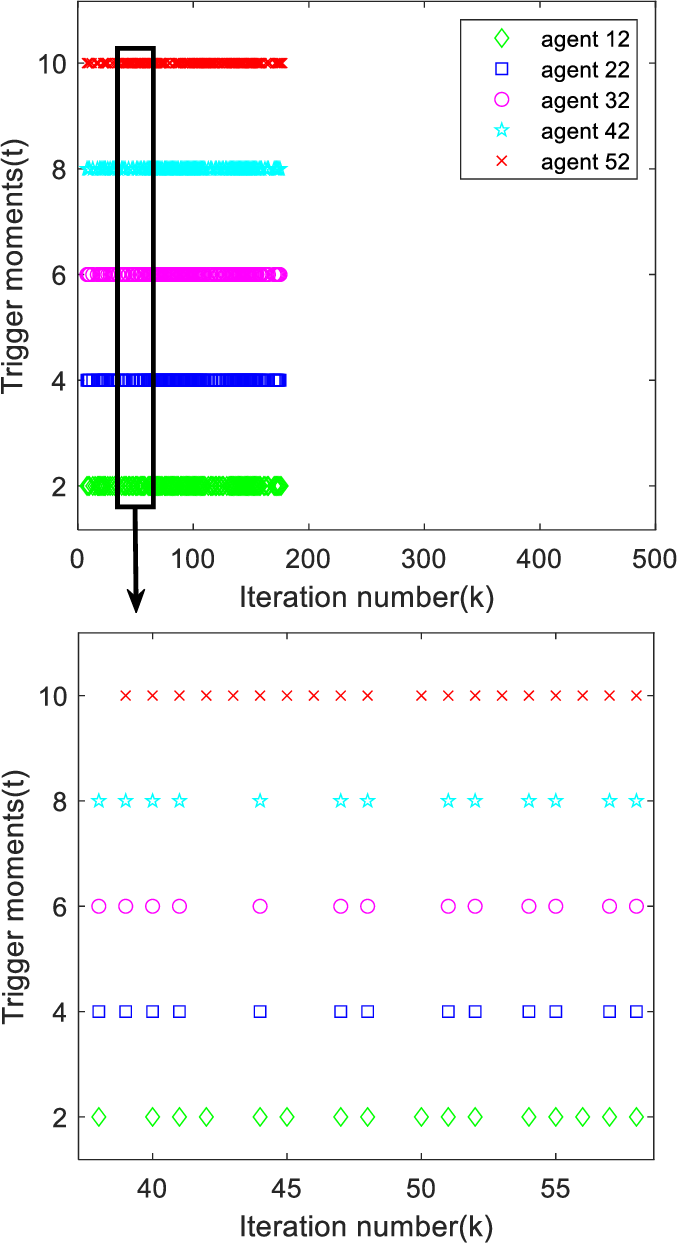

Supplement: S1 Data — (ZIP) [file pone.0315209.s001.zip › S1 data/Figures/Fig 14.tif]

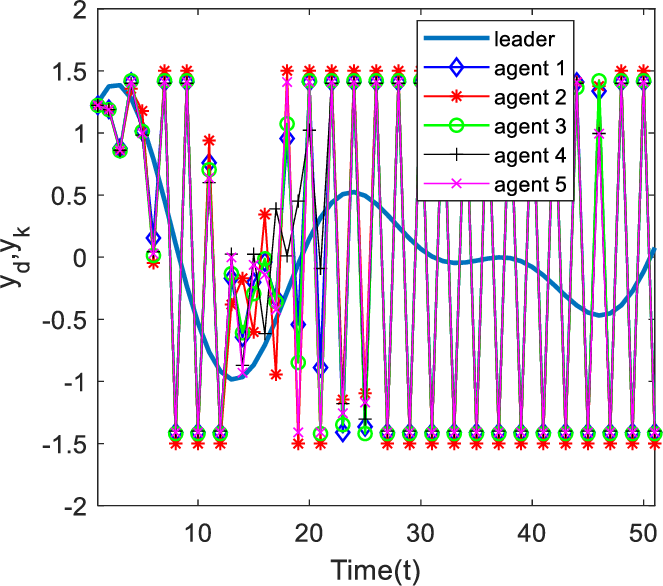

Supplement: S1 Data — (ZIP) [file pone.0315209.s001.zip › S1 data/Figures/Fig 2.tif]

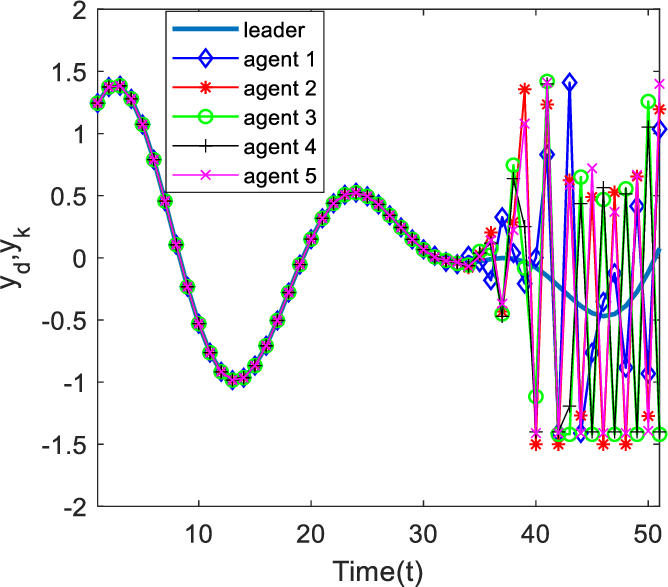

Supplement: S1 Data — (ZIP) [file pone.0315209.s001.zip › S1 data/Figures/Fig 3.tif]

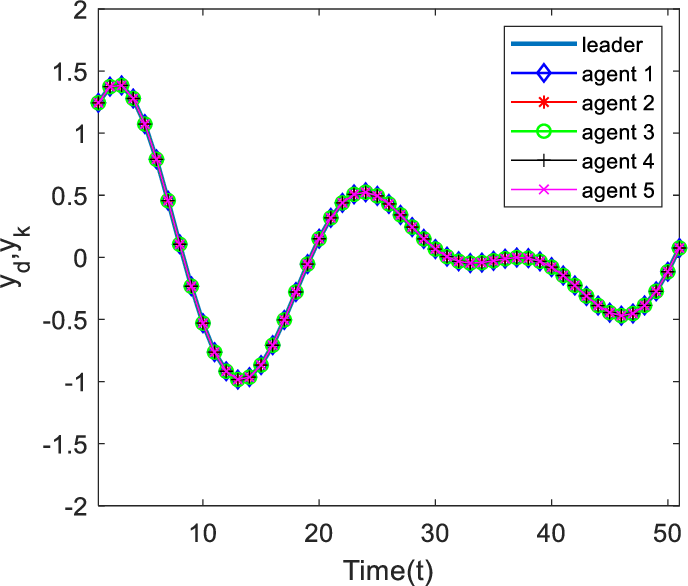

Supplement: S1 Data — (ZIP) [file pone.0315209.s001.zip › S1 data/Figures/Fig 4.tif]

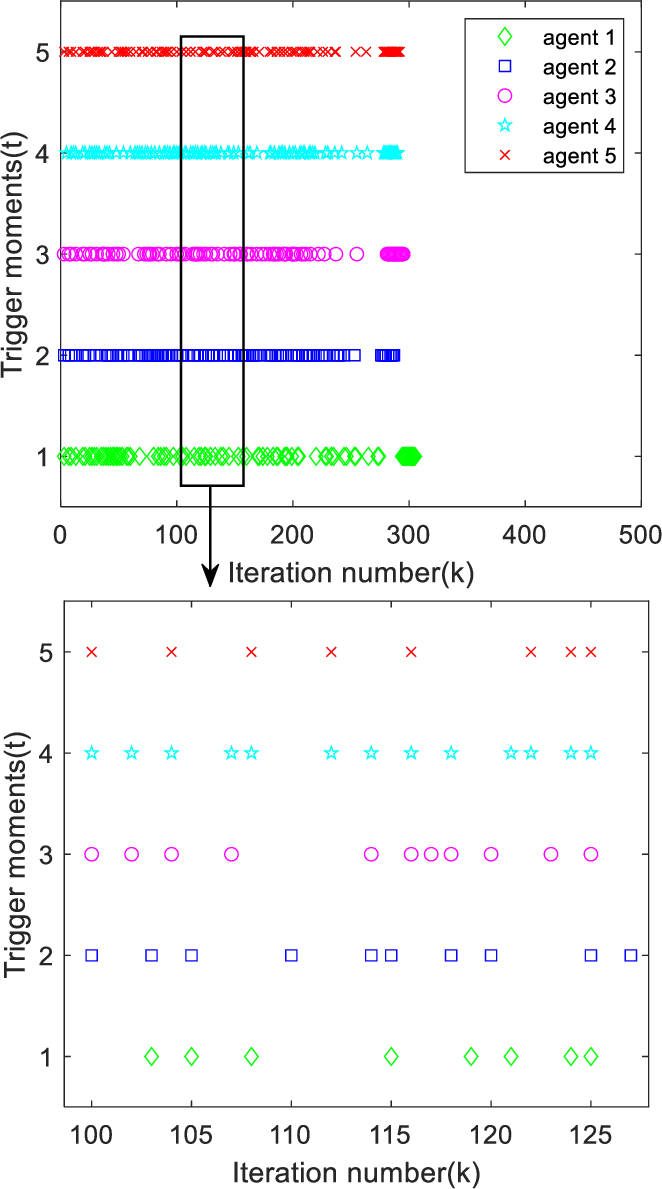

Supplement: S1 Data — (ZIP) [file pone.0315209.s001.zip › S1 data/Figures/Fig 5.tif]

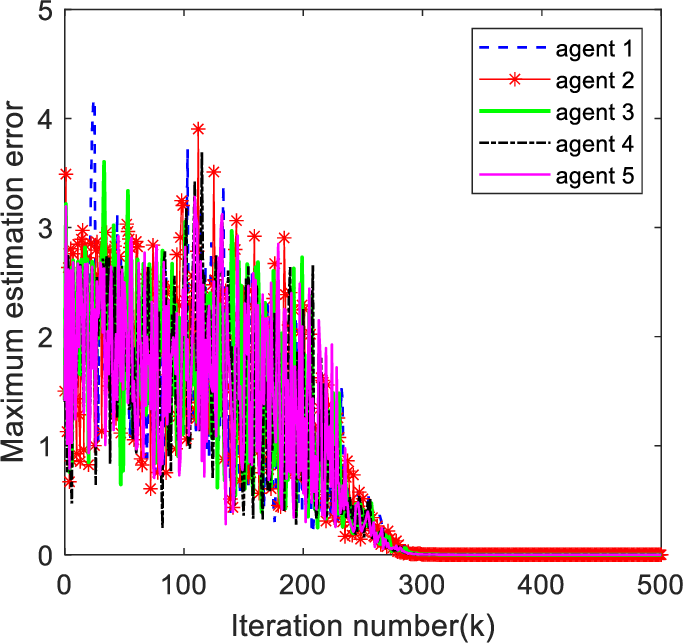

Supplement: S1 Data — (ZIP) [file pone.0315209.s001.zip › S1 data/Figures/Fig 6.tif]

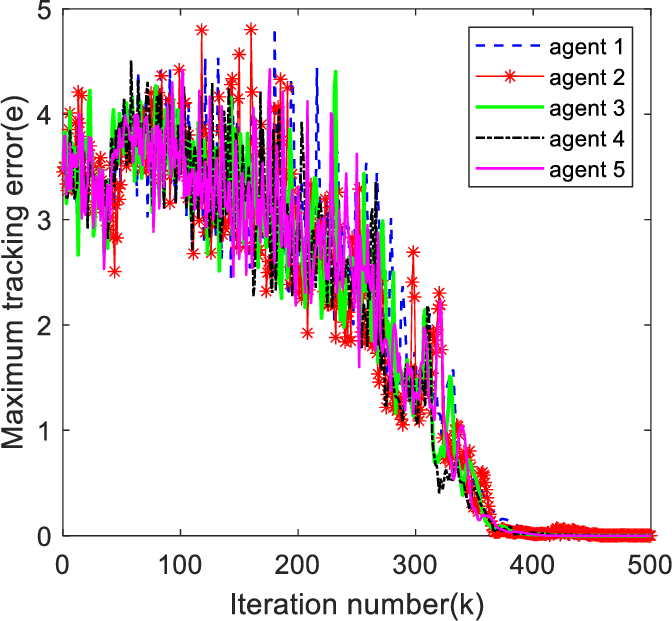

Supplement: S1 Data — (ZIP) [file pone.0315209.s001.zip › S1 data/Figures/Fig 7(a).tif]

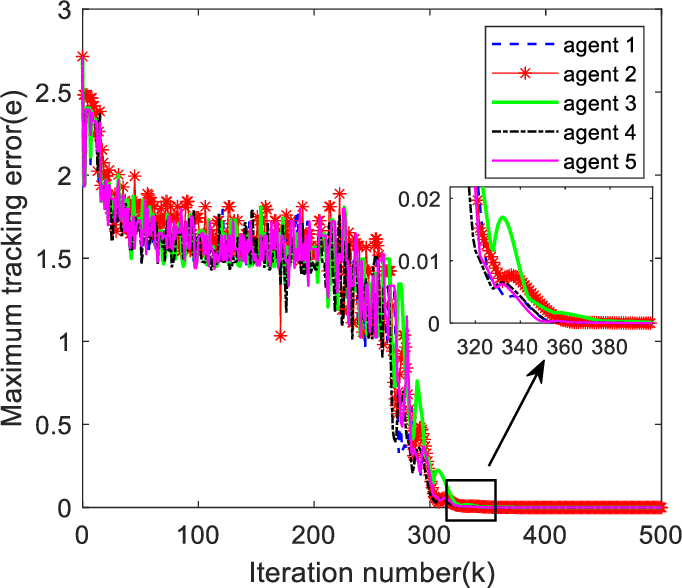

Supplement: S1 Data — (ZIP) [file pone.0315209.s001.zip › S1 data/Figures/Fig 9(b).tif]

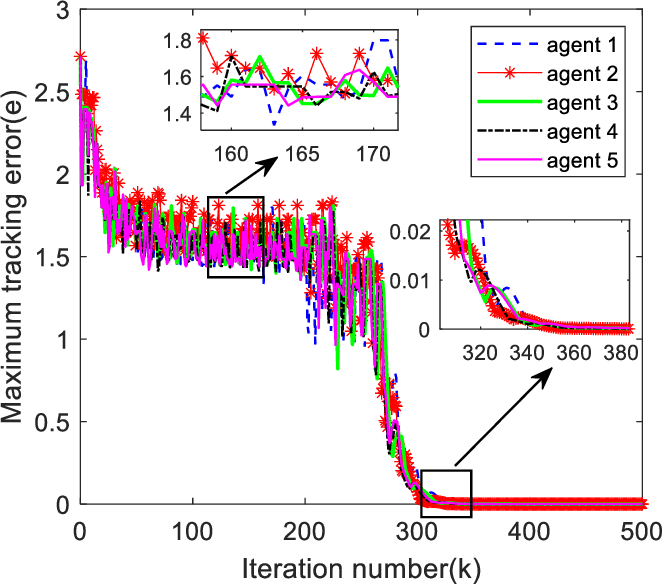

Supplement: S1 Data — (ZIP) [file pone.0315209.s001.zip › S1 data/Figures/Fig 7(c).tif]

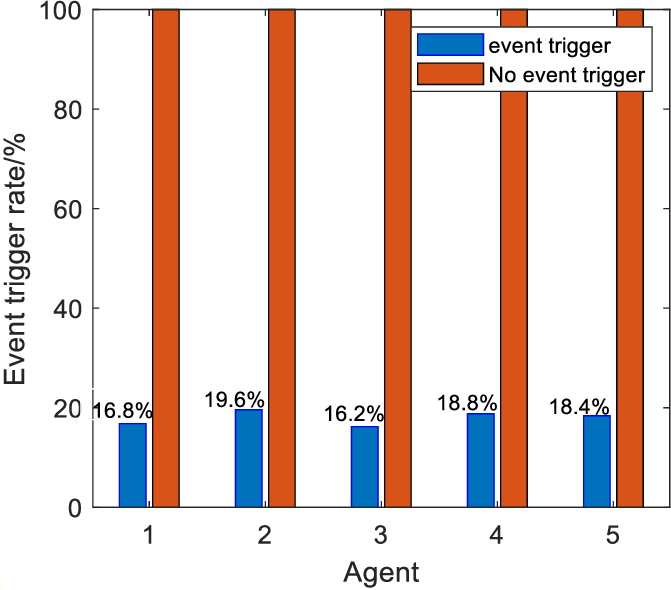

Supplement: S1 Data — (ZIP) [file pone.0315209.s001.zip › S1 data/Figures/Fig 8.tif]

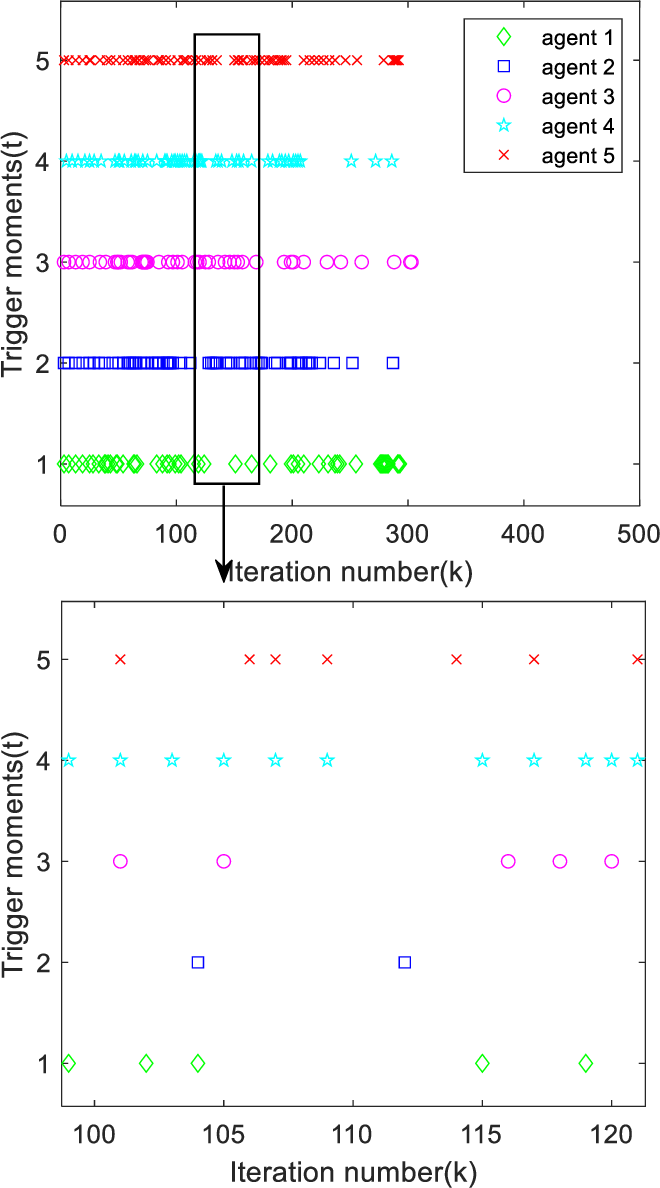

Supplement: S1 Data — (ZIP) [file pone.0315209.s001.zip › S1 data/Figures/Fig 9(a).tif]
